# Non-Toxic and Ultra-Small Biosilver Nanoclusters Trigger Apoptotic Cell Death in Fluconazole-Resistant *Candida albicans* via Ras Signaling

**DOI:** 10.3390/biom9020047

**Published:** 2019-01-30

**Authors:** Prateeksha, Braj Raj Singh, Vijai Kumar Gupta, Farah Deeba, Rajesh Bajpai, Vivek Pandey, Alim H. Naqvi, Dalip Kumar Upreti, Nicholas Gathergood, Yueming Jiang, Hesham A. El Enshasy, Essam Nageh Sholkamy, Ashraf A. Mostafa, Abd El-Latif Hesham, Brahma N. Singh

**Affiliations:** 1 Herbal Nanobiotechnology Lab, Pharmacology Division, CSIR-National Botanical Research Institute, Lucknow 226001, India; pratikschemistry@gmail.com; 2 Centre of Excellence in Materials Science (Nanomaterials), Z.H. College of Engineering and Technology, Aligarh Muslim University, Aligarh 202002, India; brajviro@gmail.com (B.R.S.); aligarhnano@gmail.com (A.H.N.); 3 ERA Chair of Green Chemistry, Department of Chemistry and Biotechnology, Tallinn University of Technology (TUT), 12618 Tallinn, Estonia; nicholas.gathergood@ttu.ee; 4 Plant Ecology and Climate Change Science Division, CSIR-National Botanical Research Institute, Lucknow 226001, India; farahnbri@gmail.com (F.D); v.pandey@nbri.res.in (V.P.); 5 Lichenology Laboratory, Plant Biodiversity and Conservation Biology Division, CSIR-National Botanical Research Institute, Lucknow 226001, India; bajpaienviro@gmail.com (R.B.); upretidknbri@gmail.com (D.K.U.); 6 Key Laboratory of Plant Resource Conservation and Sustainable Utilization, South China Botanical Garden, Chinese Academy of Sciences, Guangzhou 510650, China; ymjiang@scbg.ac.cn; 7 Institute of Bioproduct Development, Universiti Teknologi Malaysia, Johor Bahru 81310, Malaysia; henshasy@ibd.utm.my; 8 Department of Botany and Microbiology, College of Science, King Saud University, P.O. Box 2455, Riyadh 11451, Saudi Arabia; essam_92003@yahoo.com (E.N.S.); ashraf812@yahoo.com (A.A.M.); 9 Meta-Genome Biotechnology, Genetics Department, Faculty of Agriculture, Assiut University, Assiut 71526, Egypt; hesham_egypt5@aun.edu.eg

**Keywords:** fluconazole-resistant *Candida albicans*, biosilver nanoclusters, apoptosis, Ras signaling pathway, oxidative stress, proteomics

## Abstract

Silver-based nanostructures are suitable for many biomedical applications, but to be useful therapeutic agents, the high toxicity of these nanomaterials must be eliminated. Here, we biosynthesize nontoxic and ultra-small silver nanoclusters (rsAg@NCs) using metabolites of usnioid lichen (a symbiotic association of algae and fungi) that exhibit excellent antimicrobial activity against fluconazole (FCZ)-resistant *Candida albicans* that is many times higher than chemically synthesized silver nanoparticles (AgNPs) and FCZ. The rsAg@NCs trigger apoptosis via reactive oxygen species accumulation that leads to the loss of mitochondrial membrane potential, DNA fragmentation, chromosomal condensation, and the activation of metacaspases. The proteomic analysis clearly demonstrates that rsAg@NCs exposure significantly alters protein expression. Most remarkable among the down-regulated proteins are those related to glycolysis, metabolism, free radical scavenging, anti-apoptosis, and mitochondrial function. In contrast, proteins involved in plasma membrane function, oxidative stress, cell death, and apoptosis were upregulated. Eventually, we also established that the apoptosis-inducing potential of rsAg@NCs is due to the activation of Ras signaling, which confirms their application in combating FCZ-resistant *C*. *albicans* infections.

## 1. Introduction

At the beginning of the 20th century, infectious diseases were the principal cause of death worldwide [1]. The decrease in morbidity and mortality from microbial infections over the last century was attributed mainly to the development of a novel class of silver-based antimicrobial agents [2]. When the era of antibiotics began with the discovery of penicillin, the use of silver abated. However, due to the emergence of multi-drug resistance (MDR) in microorganisms to conventional agents [3], the use of silver for treating infections has regained importance [4]. Nevertheless, the clinical application of silver ions has one major disadvantage; they are easily inactivated by complexation and precipitation. Thus, the use of silver ions has been restricted [5]. Owing to their intrinsic low toxicity and high translational usage, silver nanoclusters (Ag-NCs) have great potential for combating infectious diseases that are caused by the various genera of the pathogenic microorganisms [6,7,8]. *Candida albicans*, an important human fungal pathogen, is related to an array of clinical conditions, ranging from irritating superficial infections of the vaginal and oral mucosa, to life-threatening systemic disease in immune-compromised patients [9,10]. *Candida* infections are often recalcitrant to therapy and have developed MDR to traditional therapeutic agents [11]. Searching for more effective antifungal therapies is therefore of paramount importance. Specifically, understanding the mechanistic basis of cell death decisions in *C. albicans* may well provide new developments in the search for novel antifungal agents.

Owing to their new or improved properties and high translational value, silver nanostructures have great potential for their use in healthcare products [12,13]. In comparison to metal nanoparticles (NPs), nanoclusters (NCs) with superior reactive oxygen species (ROS) producing capacity are highly sought after, because of their outstanding antimicrobial and anticancer activities [2,14,15,16,17,18]. However, chemically synthesized Ag-NCs have significant and challenging toxicity issues that limit their applicability as promising antimicrobial agents [19]. The use of chemicals, high temperature, and pressure have been proposed as a means to synthesize Ag-NCs for antimicrobial applications [20]. Unfortunately, the physico-chemical route often yields hazardous by-products for polluting the environment, restricting the development of safe nanomaterials for biomedical applications. Higher reactivity also remains a concern for biomedical applications [6]. To date, numerous investigations have been conducted that focus on utilizing natural metabolites to synthesize the desired nanomaterials for various biomedical applications [6,12,21,22,23,24,25,26]. Drug resistance in *C. albicans* during fluconazole (FCZ)-mediated chemotherapy is a major barrier for successful candidiasis treatment. Thus there is an urgent need for new antifungal agents with improved efficacy against drug resistant *C*. *albicans* [1,3,10]. Several studies demonstrating the anticandidal activity of silver-based nanomaterials are available [15,27,28]. However, it remains unclear as to whether silver-based biomaterials could target the cellular signaling pathway of *C. albicans* to exhibit their anticandidal activity. To overcome toxicity challenges, we demonstrated the fabrication of stabilized and safe biosilver nanoclusters (rsAg@NCs), using a metabolite-rich extract of the usnioid lichen “*Usnea longissima*” (a symbiotic association of algae and fungi) that efficiently inhibited the growth of FCZ-resistant *C*. *albicans*, rather than chemically synthesized AgNPs (cs-AgNPs). Eventually, we also elucidated the molecular mechanisms of action of the rsAg@NCs for inducing apoptotic cell death in FCZ-resistant *C*. *albicans*.

## 2. Materials and Methods

### 2.1. Biosynthesis and Characterization of rsAg@NCs

A total of 90 mL of 3 mM silver acetate was placed in a culture flask. Under magnetic stirring, 10 mL of distilled water (DW) containing 2 mg of aqueous extract of *U. longissima* (AEU) was added to the Ag^+^ solution. After the addition of AEU, the pH value of the mixture was immediately adjusted to ~10 by adding 1 M NaOH solution. Furthermore, 5 units/mL of polyphenol oxidase (PPO) was mixed with Ag^0^ solution for the bioconversion of polyphenols into *o*-quinones. The color of the mixture rapidly changed from colorless to brown, indicating the formation of Ag-NCs, which are denoted as “rsAg@NCs”. 

UV-Vis absorbance spectra at different time intervals were recorded using a Perkin-Elmer Lambda-45 spectrophotometer (Waltham, MA, USA). The MiniFlex™ II benchtop X-ray powder diffraction (XRD) system (Rigaku Corporation, Tokyo, Japan) was employed to provide XRD of the NCs, using nickel-filtered Cu Kα radiation λ = 1.5406° A at 40 kV and 30 mA. The diffracted intensities were recorded from 20° to 80° 2θ angles. The rsAg@NCs solution was centrifuged at 20,000 rpm for 20 min, and the pellet was redispersed in milli water to get rid of any uncoordinated biomolecules. The process of centrifugation and redissolving in MQ water was repeated three times to ensure better separation of free entities from the rsAg@NCs. The purified pellets were dried, grained, and then subjected to Fourier-transform infrared spectroscopy (FTIR) measurement. The measurements were performed on a Perkin Elmer FTIR spectrometer BX (Shelton CT, USA) with a solid potassium bromide method, with 4 cm^−1^ resolution and 10 scanning times. Measurements were carried out in the range of 500 cm^−1^ to 3000 cm^−1^ to determine the functional groups on the NCs.

Thermogravimetric analysis (TGA) was performed by Sieco SII, SSC5100 (IVIUM Technologies, Netherland). Dried samples (10.5 mg) were placed in the TGA furnace, and the measurements were carried out at 10 °C/min under a nitrogen atmosphere. Transmission electron microscopy (TEM) micrographs were recorded using a JSM- 1200 EX-II, JEOL equipped with an electron diffraction pattern. The mean diameter of the NCs was measured from the images obtained by TEM. Samples were prepared by depositing a drop of colloidal solution onto carbon-coated copper grids and drying at room temperature. Elemental composition of the sample was analyzed on the Oxford Instruments INCAx-sight EDAX spectrometer coupled to the scanning electron microscope (SEM). All electrochemical experiments were carried out in a 10 mL voltammetric cell at room temperature, using a three-electrode configuration, including a glassy carbon electrode (GCE; 3 mm), a working electrode, an Ag/AgCl reference electrode, and the platinum wire auxiliary electrode. Unless otherwise stated, all measurements were performed at a 50 mV/s scan rate. The measurements were carried out in 50 mM phosphate buffer (pH 7.4) containing with/or without chloride ions.

### 2.2. Assessment of Anti-Candidal Activity of rsAg@NCs

Fluconazole-resistant *C. albicans* NBC099 was maintained at 37 °C by biweekly transfer onto a fresh slant of SG agar (glucose 40 g/L, mycological, peptone 10 g/L, and agar 15 g/L). For experimental use, a small colony was picked up from the agar slant through pipetting, and the yeast cells were washed with Dulbecco’s phosphate-buffer saline (PBS) by centrifugation at 1500 rpm for 5 min. The cells were suspended in SG broth medium.

The clonogenic assay was used to examine the anticandidal activity of rsAg@NCs. Briefly, a 5 mL active culture of *C. albicans* (1 × 10^10^ cells/mL) was centrifuged at 5000 rpm for 5 min at 4 °C. Then, the pellet was washed with PBS and resuspended in PBS. One hundred microliters of suspended cells were dispensed into the 96-well microtiter plate in triplicates and test drugs diluted in 100 µL sterile sabouraud dextrose (SD) broth medium was added. The plates were incubated at 37 °C for 2 h. The whole suspension of the plate wells was spread on the SG agar plate and incubated at 37 °C for 24 h. Anticandida activity of rsAg@NCs was determined by counting the colony forming units (cfu)/mL.

The agar disc diffusion assay was also employed to assess the antifungal activity of rsAg@NCs. One hundred microliters of suspended cells were spread uniformly on SG agar plates, and test drugs were loaded onto the pre-sterilized filter paper disc. The Petri plates were incubated at 37 °C for 24 h. The zone of inhibition was determined through the measurement of the diameter of *C. albicans* cell clearance around the disc.

### 2.3. Live and Dead Cell Staining Assay

Cells were seeded on a glass cover slip for 12 h in SG broth medium at 37 °C, and then treated with test drugs. After 24 h, cell viability was examined by fluorescence microscopy. Cell viability was assessed using the LIVE/DEAD cell viability staining kit (Carlsbad, CA, USA). Cells were prepared for analysis as per the manufacturer’s protocol. Live SYTO 9-stained green cells and dead propidium iodide (PI)-stained red cells were analyzed by the Cellinsight CX7 fluorescence microscope (Thermo Fisher, USA).

### 2.4. Cell Viability Assay

The tetrazolium dye, 3-(4,5-dimethylthiazol-2-yl)-2,5-diphenyltetrazolium bromide) tetrazolium (MTT), was used to assess the viability of *C*. *albicans* NBC099. An aliquot of cell suspension (1 × 10^6^) was seeded in a 96-well microtiter plate, treated with test drugs, and incubated for 24 h at 37 °C. After washing the cells with PBS, 100 µL of glucose-free RPMI-1640 medium containing MTT (0.5 mg/mL) was added to each well and incubated for 4 h at 37 °C. The culture medium was aspirated, and the cells were incubated for 15 min with 100 µL of acidic isopropanol (0.09 N HCl) to dissolve the formazan crystals. The absorbance of the MTT formazan was recorded at A_570_ using a microplate reader (Bio-Rad laboratories Inc., Hercules, CA, USA).

### 2.5. Mitochondrial Membrane Potential (MMP) Assay

Mitochondrial membrane potential (MMP) was measured by using a JC-1 dye as a molecular probe, as described elsewhere [29]. MMP was also analyzed by using rhodamine-123 (Sigma Aldrich) staining fluorescence dye. Cells were washed twice with prewarmed PBS (37 °C) and stained with 5 μM of rhodamine-123 for 15 min at 37 °C. Afterwards, fluorescence imaging was carried out on a Cellinsight CX7 fluorescent microscope (Thermo Fisher, USA) to examine the changes in MMP of *C. albicans* NBC099.

### 2.6. Apoptosis Assays

Immunofluorescence detection of apoptosis by rsAg@NCs was used. Briefly, cells (2 × 10^8^ cfu/mL) were seeded in eight-chamber slides (Nunc-Labtek, IL, USA) in SG broth media containing test drugs, and incubated for 24 h at 37 °C. Following the treatment, the cells were washed with PBS thrice, and incubated with CK18 monoclonal fluorescence antibody (1:80), and the cells were analyzed by a Cellinsight CX7 fluorescence microscope (Thermo Fisher, USA) using an M30 assay kit (Roche Applied Sciences, Germany).

Terminal deoxynucleotidyltransferase dUTP nick-end labeling (TUNEL) is a method for detecting DNA fragmentation by labeling the terminal ends of nucleic acids. DNA strand breaks in candidal cells were analyzed by TUNEL assay [30]. Untreated or treated cells were washed twice in PBS and then fixed in 4% paraformaldehyde for 24 h at 4 °C. Fixed cells were washed twice in PBS. Cell permeabilization and the TUNEL reaction were carried out, using the APO-BrdU TUNEL assay kit (Thermo Fisher, USA) as described in the manufacturer’s protocol. Induction of apoptosis was analyzed by fluorescence microscope and flow cytometry.

Nuclear condensation and fragmentation were assessed by using the DAPI (4′,6-diamidino-2-phenylindole) staining assay. Cells were grown on glass cover slides and treated with rsAg@NCs for 24 h. The collected cells were washed twice with PBS and permeabilized in a permeabilization solution (0.1% Triton X-100 and 0.1% sodium citrate). Again, cells were washed with PBS and incubated with 1 µg/mL of DAPI in the dark for 30 min at 37 °C. To examine nuclear staining, cells were examined by an EVOS fluorescence microscope (Invitrogen, USA).

Apoptosis was also examined by fluorescence microscopy, using acridine orange (AO). After fixation of cells in 4% paraformaldehyde solution at 4 °C overnight for permeabilization, cells were incubated in 5 µL/mL of AO for 10 min at 37 °C. Detection of apoptosis was recognized by shrunken cells, fragmented nuclei, and fluorescence enhancement by condensed chromatin. The cells in the final state of apoptosis turned orange.

### 2.7. Analysis of Oxidative Stress Markers

The H_2_DCFDA staining assay was applied to detect the accumulation of intracellular reactive oxygen species (ROS). Cells grown on glass cover slides and seeded in 96-well microtiter plates for fluorescence microscopy and microplate reader, respectively and incubated at 37 °C for 24 h. Cells were washed with PBS and treated with 5 mM of H_2_DCFDA for 30 min at 4 °C. Cells were washed twice, and immediately examined by fluorescence microscopy, or absorbance was recorded for total fluorescence at an excitation wavelength of 485 nm, and an emission wavelength of 520 nm, using a microplate reader (Thermo Fisher, USA).

The content of hydroxyl-free radicals (^•^OH) was examined in the cell hydrolysate of *C. albicans* NBC099, using a sonication method. The reaction mixture (3.0 mL) contained 80 mM potassium phosphate buffer (pH 7.4), 10 mM sodium pyrophosphate, 10 mM MgCl_2_, 0.3 mM NADP^+^, 8 mM glucose 6-phosphate, seven units of glucose 6-phosphate dehydrogenase, and 5 mg of protein hydrolysate. Reactions were initiated by the addition of NADP^+^. Reactions were carried out at 37 °C in a center-well flask containing 0.6 mL of 15 mM semicarbazide hydrochloride in 150 mM potassium phosphate (pH 7.4). The sealed flask was incubated overnight at room temperature. An aliquot (0.2 mL) was diluted to 3.0 mL with DW, and the absorbance at 224 nm was recorded.

To measure the hydrogen peroxide (H_2_O_2_) scavenging activity, *C. albicans* NBC099 cells were cultured with or without rsAg@NCs in the logarithmic phase for 24 h at 37 °C, collected by centrifugation, washed with PBS, and resuspended into PBS. H_2_O_2_ (1.5 mM) was added to the cell suspension, and then the mixture was incubated for 30 min at 37 °C. Next, 60 µL of 0.25 M Amplex Red reagent (Invitrogen, USA) and 60 µL of horseradish peroxidase (0.02 mg/mL) were added. The intensity of the fluorescence generated was measured using a microplate reader (Thermo Fisher, USA).

### 2.8. Extraction and Identification of *C. albicans* Proteins for Proteome Analysis

The culture of *C. albicans* NBC099 was washed thrice with PBS and lyophilized until the pellets become dry. The dried pellets were crushed in liquid N_2_, mixed with 1 mL of extraction buffer [(50 mM Tris-HCl pH 8.0, 25 mM EDTA, 500 mM thiourea, and 0.005% β-mercaptoethanol (BME)] and mixed well then added 10 mL of 10% trichloroacetic acid (TCA) in acetone with 0.007% BME. The mixture was kept at −20 °C overnight. Next, the mixture was centrifuged at 4500 rpm for 10 min at 4 °C. The supernatant was discarded, and the obtained pellets were washed with acetone and 0.007% BME solution. The pellets were dried and lyophilized for 30 min. Then, the dried pellets were mixed with rehydration buffer (7 M urea, 2 M thiourea, 2% CHAPS (3-[(3-Cholamidopropyl)dimethylammonio]-1-propanesulfonate), 0.5% ampholyte and 50 mM DTT), and dissolved for 3 h with shaking at 20 °C. The total protein concentration was quantified by the Bradford Assay (Bio-Rad, Hercules, CA, USA), using bovine serum albumin (BSA) as the standard.

To compare the proteomes of the rsAg@NCs-treated cells lysate with a hose of untreated cells lysate, two-dimensional gel electrophoresis and mass spectrometry were applied. The extracted cytosolic proteins were subjected to isoelectric focusing (IEF) in the first dimension, followed by sodium dodecyl sulfate polyacrylamide gel electrophoresis (SDS-PAGE) as the second dimension, according to the methods of Deeba et al. [31]. Gels non-destructively stained with silver stain kit (Bio-Rad) and were scanned (resolution, 300 dots per inch). The gel images were analyzed with Image Master 2D platinum software (v 7.0; GE Healthcare, Little Chalfont, UK) to determine their differential expression of proteins. The analyses were done on the basis of the % volume values of the spots exhibiting 1.5-fold differential expression (either positive or negative) as compared to the control spots, with p-values under 0.05. Three technical replicates were run for each biological sample of *C. albicans*. The spots were cut and digested with trypsin overnight at 37 °C. Proteins were identified by using matrix-assisted laser desorption ionization -time of fight (MALDI/TOF)-TOF), as previously described [32].

### 2.9. ROS Detection Assay

ROS generation was also assessed through the incubation of cells with dihydrorhodamine (DHR)-123 (5 µg/mL) for 30 min before the end of each experiment. Cells were washed twice with ice-cold PBS and analyzed by flow cytometry.

### 2.10. Measurement of Endogenous Antioxidants

The cell lysate of untreated or rsAg@NCs-treated candidal cells was prepared and used for the detection of endogenous antioxidants. A method of Ohkawa et al. [33] was used to measure the lipid peroxide. Activities of catalase (CAT) and superoxide dismutase (SOD) were measured according to the methods of Sinha [34] and Kakkar et al. [35], respectively. Intracellular reduced (GSH) and oxidized (GSSG) glutathione levels were measured according to the method of Tiezte [36]. Protein concentrations were determined by using the Bio-Rad protein assay kit (Bio-Rad laboratories, CA, USA).

### 2.11. Detection of AhpC Expression

Total RNA was isolated using the QiagenRNeasy Mini Kit according to the manufacturer’s protocol (Qiagen, Valencia, CA, USA). Ten nanograms of purified RNA were loaded into each well of a formaldehyde–agarose gel. After electrophoretic separation, RNA was transferred to a piece of nylon membrane and hybridized with radioactively labelled AhpC DNA probe, prepared as previously described [37]. Prehybridization, hybridization, and high stringency washes were done according to Mongkolsuk et al. [37].

### 2.12. Statistical Analysis

The data were analyzed to obtain mean values and standard errors (SE) for all treated and untreated control samples, which were subjected to statistical comparison by using the Student *t*-test, where *p* < 0.001 was considered as being significant using SPSS version 21.

## 3. Results

### 3.1. Biosynthesis of rsAg@NCs and Their Characterization

**Figure 1:**
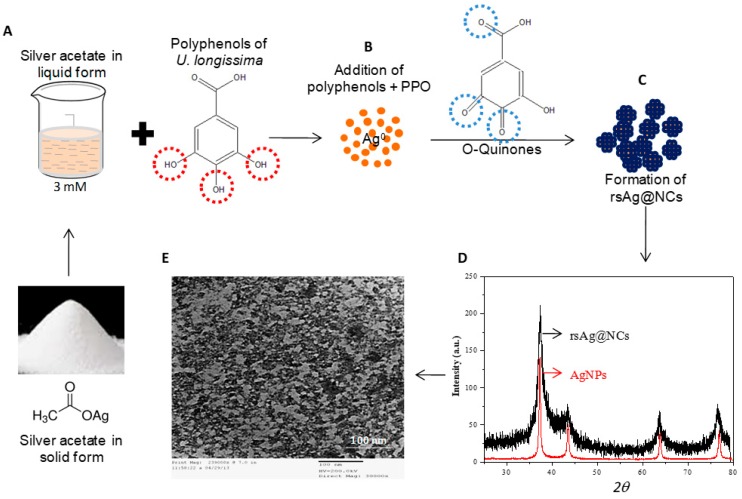
Approach to for fabricating bionanoclusters of silver (rsAg@NCs) using phytochemicals of *Usnea longissima*. (**A**) A solution of silver acetate (3 mM) was challenged with aqueous extract of *U. longissima* (AEU; 2 mg/mL) in reaction glassware. (**B**), Polyphenols of AEU reduce Ag^+^ into Ag°, and form *o*-quinones, due to bioconversion of polyphenols by the action of polyphenol oxidase (PPO; 5 units/mL). Quinones contain an oxygen group (neutral charge), and an accumulation of quinones on the surface of AgNPs helps to form (**C**), clusters of AgNPs (denoted as “rsAg@NCs) due to the absence of electronic hindrances between the nanoparticle (NPs). (**D**) x-ray diffraction (XRD) pattern of the rsAg@NCs and AgNPs were recorded in the range of 20° to 80° at a 2θ angle. XRD pattern of the rsAg@NCs depicts the well-resolved diffraction peaks broadening of the crystalline zerovalent silver (Ag°) NCs as compared to the AgNPs, which can be assigned to the reflection planes: 111, 200, 220, and 311. (**E**) Transmission electron microscopy(TEM) image reveals the shape, size, and clustering nature of the NCs. Red circles show the hydroxyl groups of the polyphenols, which are responsible for reducing the potential by donating hydrogen to Ag^+^ and forming Ag°. Blue circles are indicating neutral charge-free oxygen molecules (*O*-quinones) by the action of polyphenol oxidase (PPO) that promote the formation of NCs of AgNPs.

There are no previous reports on the expeditious and green synthesis of Ag-NCs using the aqueous extract of *U. longissima* (AEU). AEU contained a high content of polyphenolic compounds, and exhibited a promising reduction potential (Figure S1A–D), indicating the presence of phytochemicals in AEU that act as an excellent template for the synthesis of AgNPs. We synthesized Ag-NCs (denoted as “rsAg@NCs”) via the bio-transformation of polyphenolic metabolites of AEU into *O*-quinones by the action of polyphenol oxidase (5 units/mL) (Figure 1A–C). This process helps to synthesize NCs by modifying the AgNPs surface charge. Plant quinones contain free oxygen molecules, having a neutral charge. Subsequently, the accumulation of quinones on the surface of AgNPs might be responsible for providing a neutral charge to the NPs, and increase their interaction, thereby forming NCs (Figure 1C). The dark brown color of the rsAg@NCs at pH ~10 was observed, and the rapid synthesis of NCs was confirmed by UV-Vis spectrophotometry. The appearance of color was due to the accumulation of localized surface plasmon resonance (LSPR) (Figure S2A), which are typical of Ag nanomaterials having λ_max_ values that are reported in the visible range of 400–450 nm. UV spectra clearly exhibited the LSPR band of an Ag-based nanomaterial at 340 nm [38]. The optical band gap of rsAg@NCs was determined by the Tauc relation, αhν = A(hν − Eg)^n^. The measured band gap of the rsAg@NCs was found to be ~3.3 eV (Figure S2B), which may be attributed to the quantum confinement effect. The synthesis of the NCs was completed in 2 min, and they settled down at the bottom within 5 min through electron conduction in both the ground and excited states, which is confined to dimensions that are smaller than the electron mean free path [39]. To determine the optimum concentration of AgNO_3_, the mixture containing rsAg@NCs was centrifuged at 5000 rpm for 10 min. The obtained supernatant was again used for the fabrication of rsAg@NCs, following the same method applied previously. An addition of the same amount of AgNO_3_ did not change the color of the supernatant. These results suggest that the 3 mM concentration of AgNO_3_ is optimum for the synthesis of rsAg@NCs by the desired amount of AEU.

The crystalline nature of rsAg@NCs was confirmed by XRD. Figure 1D reveals the XRD pattern of rsAg@NCs, which had four Bragg reflections that could be indexed on the basis of the face-centered cubic (fcc) structure of silver. A comparison of the XRD spectrum with the standard JCPDS file (04-0783) confirmed the presence of silver in the form of an fcc crystalline lattice, as evident from the peaks at 2θ values of 37.31°, 43.51°, 63.68°, and 76.43°, representing the (111), (200), (220), and (311) Bragg’s reflections of the fcc structure of silver, respectively. The average crystalline size was calculated from the full width at half-maximum of the diffraction peak (111) using the Scherrer equation, and it was found to be ~2.8 nm, suggesting that the NCs are highly anisotropic.

The size and shape of the NPs prepared by using phytochemical-rich AEU were determined by using TEM. Figure 1E shows the TEM image of the rsAg@NCs, depicting the lattice fringes clearly. In addition, the image revealed spherical-shaped NPs, and the sizes of the particles ranged between 2–4 nm, with an average size of 3 nm, which is consistent with XRD and optical results. The particles present in the cluster form may be due to the small dimensions and high surface energies of the particles. The TEM image also showed a thin outer layer around the particles, indicating the encapsulation of NPs formed by quinones.

The energy dispersive X-ray (EDX) analysis showed a strong signal of silver, and again confirmed the synthesis of AgNCs (Figure S3). Metallic Ag nanocrystals generally show a typical optical absorption peak at ~3 keV, which is characteristic for the absorption of metallic silver [6], indicating the presence of zerovalent silver in rsAg@NCs. The data confirm the successful synthesis of rsAg@NCs, using polyphenolic metabolites of AEU. The photoluminescence behavior of rsAg@NCs could give information about the energies and dynamics of photogenerated charge carriers, as well as on the nature of the emitting states. The emission spectra have broadband with a maximum of 532 nm when excited at 350 nm (Figure S4).

FTIR has become a key approach for determining the interaction between NCs and the phytochemicals of AEU, which are used for the synthesis and efficient stabilization of the nanomaterials [7]. The hydroxyl groups of plant polyphenols could be responsible for the bio-reduction of Ag^+^ ions. The FTIR spectrum shows different major peak positions at 2503, 1836, 1690, 1660, and 1601 cm^−1^ (Figure S5). The similarities between the spectra of NCs and phytochemicals, with some minor shifts in peak position, clearly point out the presence of the plant polyphenols in AEU as reducing and capping agents for the AgNPs. The peaks located at 1660 and 1601 cm^−1^ could be assigned to C=O stretching or quinone bending. The broad and intense peak at 2503 cm^−1^ corresponds to OH stretching vibrations of the phenol/carboxylic group present in AEU. The peak at 1690 cm^−1^ was assigned for the binding of free quinone with silver AgNPs. *o*-Quinones have free oxygen groups with a neutral charge, which may increase the formation of NC of AgNPs, due to the alteration of the zeta potential of the NPs. Therefore, it could be inferred that the polyphenolics and quinones of *U. longissima* are responsible for synthesizing and stabilizing the rsAg@NCs.

Thermogravimetric analysis (TGA) revealed the composition of rsAg@NCs (10.355 mg; 100%), as shown in Figure S6. The TGA curve depicted the three weight loss steps of rsAg@NCs. Weight losses were observed at ~181 °C, (1.406 mg; 13.58%), ~321 °C (0.943 mg; 9.107%), and ~824.9 °C (0.799 mg; 7.71%). This result might be initially due to the removal of H_2_O molecules, while higher temperature likely causes the volatilization and thermal degradation of lower weight phytochemicals. Taken together, the TGA curve revealed a total weight loss (3.148 mg; 37.72%). The high thermal stability of the rsAg@NCs possibly results from a strong interaction between phytochemicals and Ag-NCs, which restricts thermal motion in the rsAg@NCs.

The electrochemical properties of rsAg@NCs were examined by cyclic voltammetry (CV). The rsAg@NCs exhibited sharp redox currents (±25 μA) with a narrow voltage range at ~0.0 V versus the Ag/AgCl reference electrode (Figure S7). Oxidation and reduction potentials of the rsAg@NCs (PBS; 100 mM NaCl) were found to be 0.04 V and −0.08 V, respectively. On the other hand, when the analysis was performed in PBS solution (without NaCl), the oxidation and reduction potentials were found to be 0.35 V and −0.04 V, respectively. The data together revealed that the rsAg@NCs have high peak currents with a narrow peak width, due to the high surface area-to-volume ratio of the rsAg@NCs. The voltammetric behaviors of rsAg@NCs obtained are in good agreement with the previous report on AgNPs [40]. Significantly, the redox potential of nanomaterials has been widely utilized for the killing of microbial pathogens by inducing oxidative stress [15].

### 3.2. rsAg@NCs Inhibit the Cell Viability of Candida albicans

By using a UV-Vis spectrophotometer, absorbance was measured, to determine the stability of rsAg@NCs at room temperature, and no significant change in the absorbance was recorded for up to 72 days of incubation (Figure S8A). The stabilities of rsAg@NCs were further determined in SD culture media at 37 °C for 72 h, and no alteration was detected, such as agglomeration (Figure S8B). The stability studies clearly demonstrated that the higher stability of rsAg@NCs might be due to the accumulation of *O*-quinones on the surfaces of the NCs.

**Figure 2:**
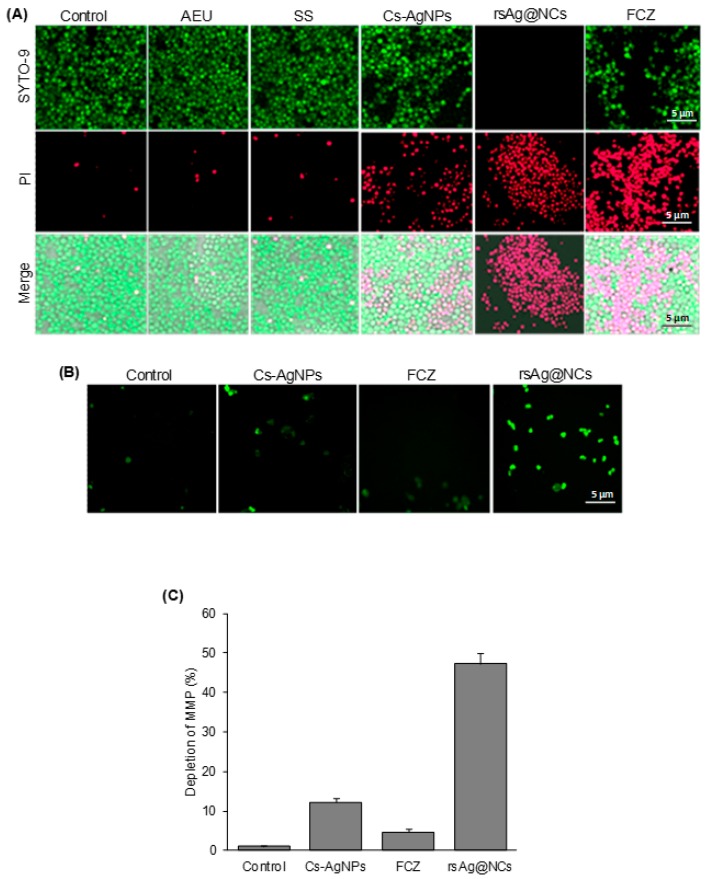
Effect of rsAg@NCs on the viability of *C. albicans* NBC099 and intracellular reactive oxygen species (ROS) accumulation. (**A**), Cells of NBC099 (2 × 10^5^/mL) were seeded on a glass cover slip in the presence of the indicated treatments: AEU (200 µg/mL), silver nitrate solution (SS; 10 µg/mL), Cs-AgNPs (10 µg/mL), FCZ (60 µg/mL), and rsAg@NCs (10 µg/mL) for 24 h at 37 °C. Cell viability was determined by using the LIVE/DEAD cell viability kit, by following the manufacturer’s protocol (Thermo Fisher, USA). SYTO-9 stained green live cells and propidium iodide (PI)-stained red cells were analyzed by a Cellinsight CX7 fluorescence microscope. (**B**), FCZ-resistant *C. albicans* NBC099 cells were seeded on glass cover slides and exposed to rsAg@NCs (10 µg/mL) for 24 h at 37 °C. After washing twice with phosphate buffer saline (PBS), the cells were incubated with 5 mM H_2_DCFDA and examined using a fluorescence microscope (**C**). The cells were also grown into 96-well microtiter plates with the above treatments, and then cells were loaded with H_2_DCFDA. ROS accumulation was subsequently analyzed by measuring fluorescence intensity at 540 nm using a microplate reader. Each reported value represents the mean ± standard error (SE) from three independent experiments (* *p* < 0.001, compared with untreated control). MMP; mitochondrial membrane potential.

The minimum inhibitory concentration (MIC) of the rsAg@NCs required to kill *C. albicans* was examined by assessing clonogenic survival. The rsAg@NCs at a concentration of 10 µg/mL were found to be fungicidal after an exposure of 24 h (data not shown). The antifungal activity of rsAg@NCs was determined by using clonogenic survival and disc diffusion assays. After incubation for 24 h, colonies of *C*. *albicans* were clearly observed to be in contact with silver acetate as well as Cs-AgNPs. However, fewer colonies were observed, when treated with the rsAg@NCs (Figure S9A). The rsAg@NCs (10 µg/mL) also exhibited a zone of inhibition (ZOI; 19.2 ± 0.5 mm), which was higher than the ZOI (4.7 ± 0.2 and 7.3 ± 0.4) formed due to the treatment of AgNPs (10 µg/mL) and fluconazole (FCZ; 60 µg/mL), respectively (Figure S9B). Exposure of the cells to rsAg@NCs revealed a significantly higher rate of cell death, as compared to Cs-AgNPs and FCZ (Figure 2A). The observed anti-candidal activity of rsAg@NCs is corroborated with studies that have been published on antimicrobial AgNPs by Hwang et al. and Monteiro et al. [15,27]. The fluorescent SYTO-9 green stain was used to examine the capacity to resist *Candida* cell attachment on rsAg@NCs (10 µg/mL) containing hydrogel-coated surfaces developed by spin-coating onto glass surfaces. Uncoated glass surfaces revealed rapid and extensive attachment of *C. albicans*, while no *C*. *albicans* was attached onto the rsAg@NCs-coated glass surfaces (Figure S10).

### 3.3. rsAg@NCs Induce Mitochondrial Dysfunction

Recently, it has been shown that the AgNPs are key factors that induce apoptosis in FCZ-resistant *C*. *albicans* NBC099. Considering the redox reaction involving silver oxidation, the redox-active mitochondria could be the likely target of rsAg@NCs. To test our hypothesis, cells were stained with rhodamine 123, and analyzed to assess the mitochondrial membrane potential (MMP) of *Candida* cells using a fluorescent microscope. The rsAg@NCs (10 µg/mL) gave a significantly higher depletion of the MMP after 24 h, as compared to Cs-AgNPs and FCZ (Figure 2B). Depletion of MMP was further assessed by flow cytometry, using JC-1 dye, which showed an inhibition of MMP of NBC099 by rsAg@NCs treatment (Figure 2C). Under analogous conditions with rsAg@NCs (10 µg/mL), but with a shorter time period of 12 h, a similar form of cell death was revealed, but to a considerably less significant degree than that observed after 24 h (result not shown).

Although the rsAg@NCs depleted MMP of candidal cells, it was still ambiguous as to how rsAg@NCs modulated mitochondrial functions. There are two major adenosine triphosphate (ATP) producing pathways in eukaryotes: one is through glycolysis, and the other is through the Krebs cycle [41]. The pathways can be examined by determining the extracellular acidification rate (ECAR; an index of the glycolytic activity) and the oxygen consumption rate (OCR; an activity level in the mitochondrial respiratory chain). Hence, ECAR and OCR levels were measured in order to verify whether rsAg@NCs affect the cell viability of *C*. *albicans* NBC099 by targeting the mitochondria, or by hindering the enzymatic redox reactions [42]. The exposure of cells to rsAg@NCs (10 µg/mL) did not affect the glycolysis status significantly until 12 h. However, there was an active burst of oxygen consumption, and the OCR deteriorated after 24 h, compared to the untreated control, Cs-AgNPs, and FCZ (Figure S11A–C). The data indicate that our rsAg@NCs target the mitochondria of FCZ-resistant *C. albicans* NBC099.

### 3.4. rsAg@NCs Enhance Intracellular ROS Accumulation

A few studies have shown that metal-based nanomaterials could trigger a burst of oxidative stress, thereby inducing a cascade of cell-death effects. A study by Cui et al. [43] revealed that the AgNPs induce cell death through the accumulation of excess ^•^OH, attributed to an oxidative damage-related pathway with the depletion of the nicotinamide adenine dinucleotide reduced- tricarboxylic acid (NADH-TCA) cycle. To investigate whether rsAg@NCs affect the cell viability of FCZ-resistant *C. albicans* NBC099 by inducing ROS formation, we performed an NAD^+^ cycling assay and determined the accumulation of ROS. We found that the rsAg@NCs elicit a significant enhancement in the NAD^+^/NADH ratio of *C. albicans* for an incubation period of up to 24 h (Figure S12).

To probe whether ROS would be generated in candidal cells from exposure to rsAg@NCs, the treated cells were loaded with the ROS measuring probe H_2_DCFDA, to analyze the ROS status by using a fluorescence microscope and a microplate reader. As shown in Figure S13A,B, 10 µg/mL of rsAg@NCs induced intracellular ROS accumulation in NBC099, as indicated by the increasing fluorescence intensity. Furthermore, the production and accumulation of ROS by rsAg@NCs was determined by using a ROS-sensitive dye, dihydrorhodamine (DHR)-123, which was oxidized to a fluorescent derivative by intracellular ROS, and their contents were quantified by flow cytometry. Cells treated with 10 µg/mL of rsAg@NCs showed time-dependent enhanced ROS accumulation, as compared to the untreated control (Figure S14). Studies in 2003 have suggested that the accumulation of ROS regulates the induction of apoptosis in yeast [44]. To examine whether the ROS induced by rsAg@NCs was related to cell damage, cells were collected after exposure to rsAg@NCs for 24 h, and observed the ultrastructure of the cells by a fluorescence microscope. As shown in Figure S15, a higher cell death rate was observed in rsAg@NCs-treated cells.

The potential of rsAg@NCs to chemically generate ROS was also examined. The iron-catalyzed Fenton reaction is known to be a promoter of free radicals under aerobic conditions. Ferritin is the iron storage protein within the cell [29]. The content of ^•^OH increased upon the incubation of cells with 10 µg/mL rsAg@NCs for 24 h (Figure S16A). With the increment of ^•^OH content, it can be hypothesized that the rsAg@NCs could transform H_2_O_2_ into ^•^OH by down-regulating alkyl hydroperoxide reductase subunit C (AhpC; a protector of cells from oxidative burst) expression, a member of the thiol-dependent peroxiredoxin family, which possesses quenching activity against H_2_O_2_ [43]. The level of AhpC after 24 h of rsAg@NCs-treated or untreated candidal cells was examined by Northern blot analysis (Figure S16B). The rsAg@NCs were found to decrease AhpC expression. The H_2_O_2_ plays a key role in the generation of ^•^OH. Therefore, we next sought to examine the concentration of H_2_O_2_, in order to confirm the inhibitory effect of rsAg@NCs on AhpC. The H_2_O_2_ quenching capacity of rsAg@NCs-treated cells decreased approximately 72% at a concentration of 10 µg/mL, as compared to untreated control cells (Figure S16C), consistent with the discovery of the down-regulation of AhpC in Northern blot analysis. These results suggest that H_2_O_2_ generation induced by rsAg@NCs accumulated in the interior of *C. albicans* cells, and most were bio-transformed into the strong oxidant ^•^OH, which could be a key factor for accelerating apoptosis by rsAg@NCs in the FCZ-resistant *C. albicans* NBC099.

The levels of oxidative stress biomarkers such as lipid peroxidation (LPO), glutathione (GSH), and antioxidant enzymes (SOD and CAT) in candidal cells were measured to gain insight into the mechanism of how rsAg@NCs modulate oxidative stress responses, resulting in their anticandidal activity. As shown in Figure S17A, there was a decrease in total GSH content, which can be considered an indication of the adaptive response of the cell to oxidative damage [29]. The oxidative state of the cells was also examined by determining the GSH/GSSG ratio, since cellular oxidative stress leads to an imbalance in GSH homeostasis. The rsAg@NCs significantly decreased the GSH/GSSG ratio (Figure S17B) compared to the control, suggesting a statistically significant degree oxidative stress in candidal cells. rsAg@NCs-induced oxidative stress was further evident via the induction of LPO (Figure S17C), and through the downregulation of SOD and CAT activities (Figure S17D,E).

### 3.5. rsAg@NCs Accelerate ROS-Mediated Apoptosis

**Figure 3:**
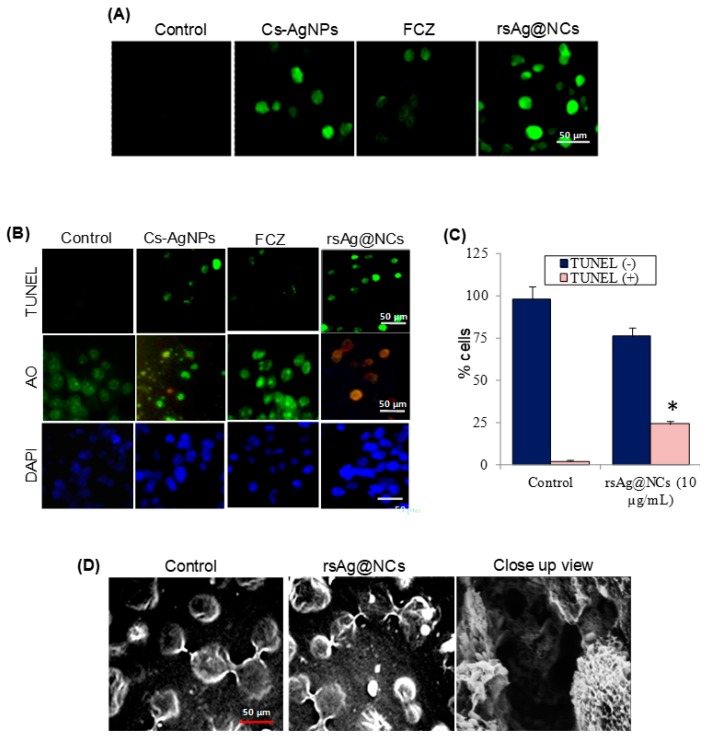
The rsAg@NCs accelerate the induction of apoptosis in FCZ-resistant *C. albicans* NBC099. (**A**) Immunofluorescence detection of apoptosis using the M30 CytoDEATH antibody that binds to the caspase-cleaved epitope of the cytokeratin 18 cytoskeletal protein, a marker of apoptosis. Cells were exposed to Cs-AgNPs (10 µg/mL), FCZ (60 µg/mL), and rsAg@NCs (10 µg/mL). After 24 h of incubation, cells were incubated with M30 CytoDEATH antibody. A marked increase in M30 fluorescence was observed by a fluorescence microscope. (**B**) Cells were treated with Cs-AgNPs (10 µg/mL), FCZ (60 µg/mL), and rsAg@NCs (10 µg/mL) for 24 h at 37 °C for further assessment of apoptosis using terminal deoxynucleotidyl transferase dUTP nick end labeling (TUNEL), acridine orange (AO), and 4′,6-diamidino-2-phenylindole (DAPI) staining assays. For the TUNEL assay, cells were washed in PBS, permeabilized for 2 min on ice and washed again with PBS. DNA ends of cells were labelled by using the APO BrdU TUNEL Assay Kit, and cells were observed under a fluorescence microscope. The permeabilized cells were stained with 5 µg/mL and 1 µg/mL of AO and DAPI, respectively. Subsequently, cells were used to detect apoptosis by using a fluorescence microscope. (**C**) The percentage of TUNEL (-) and TUNEL (+) cells due to the treatment of 10 µg/mL rsAg@NCs. Each reported value represents the mean ± SE from three independent experiments (* *p* < 0.001, compared with the untreated control). (**D**) Cells were treated with 10 µg/mL rsAg@NCs for 24 h, washed, and fixed overnight in 2.5% glutaraldehyde in phosphate magnesium buffer. Again, cells were post-fixed for 2 h in 2% osmium tetroxide. The washed cells were stained with 1% aqueous solution of uranyl acetate for 30 min. After two further items of washing, cells were dehydrated in 95% and 100% ethanol, subsequently. Cells were polymerized in 1:1 propylene epoxy embedding material (Epon) mixture, and then overnight in fresh Epon for 45 h at 65 °C. Ultra-thin sections of the cells were stained with uranyl acetate and lead citrate and examined by scanning electron microscopy (SEM).

To determine whether the rsAg@NCs could induce apoptosis events, the FCZ-resistant *C. albicans* NBC099 cells were incubated with rsAg@NCs for 24 h, and the degree of apoptotic cell death was analyzed by using the M30 CytoDEATH antibody, which binds to a caspase-cleaved epitope of the cytokeratin 18 cytoskeletal protein, a determinate maker of induction of apoptosis. As shown in Figure 3A, rsAg@NCs induced higher apoptosis, as compared to the untreated control, Cs-AgNPs, and FCZ-treated cells. To further confirm the apoptosis-inducing potential of rsAg@NCs cells, a TUNEL assay was performed to detect DNA fragmentation in NBC099. Apoptosis can be detected by labeling 3′-OH termini with modified nucleotides catalyzed by terminal deoxynucleotidyltransferase, a reliable method for the identification of apoptotic cells [44]. A strong green fluorescence or intense green fluorescent spots indicate a greater degree of breaks in the DNA nuclear strands during the later stages of apoptosis. The percentage of TUNEL-positive cells was higher when cells were exposed to the rsAg@NCs (Figure 3B,C). Acridine orange (AO) is an organic compound that emits green fluorescence when it is bound to DNA, but an orange fluorescence when it is bound to damaged DNA. Also, of interest is DAPI, which binds to the condensed nuclei of cells. Thus, both AO and DAPI can be used to examine apoptosis. Strong orange and blue fluorescence for AO and DAPI, respectively, indicated a greater degree of typical apoptotic DNA condensation and fragmentation in the nuclei of NBC099 cells exposed to rsAg@NCs, than in the intact nuclei of normal control cells (Figure 3B). However, staining was only rarely observed in untreated control cells. These findings were further supported by SEM analysis, and the formation of apoptotic bodies in rsAg@NCs-treated cells was examined (Figure 3D).

**Figure 4:**
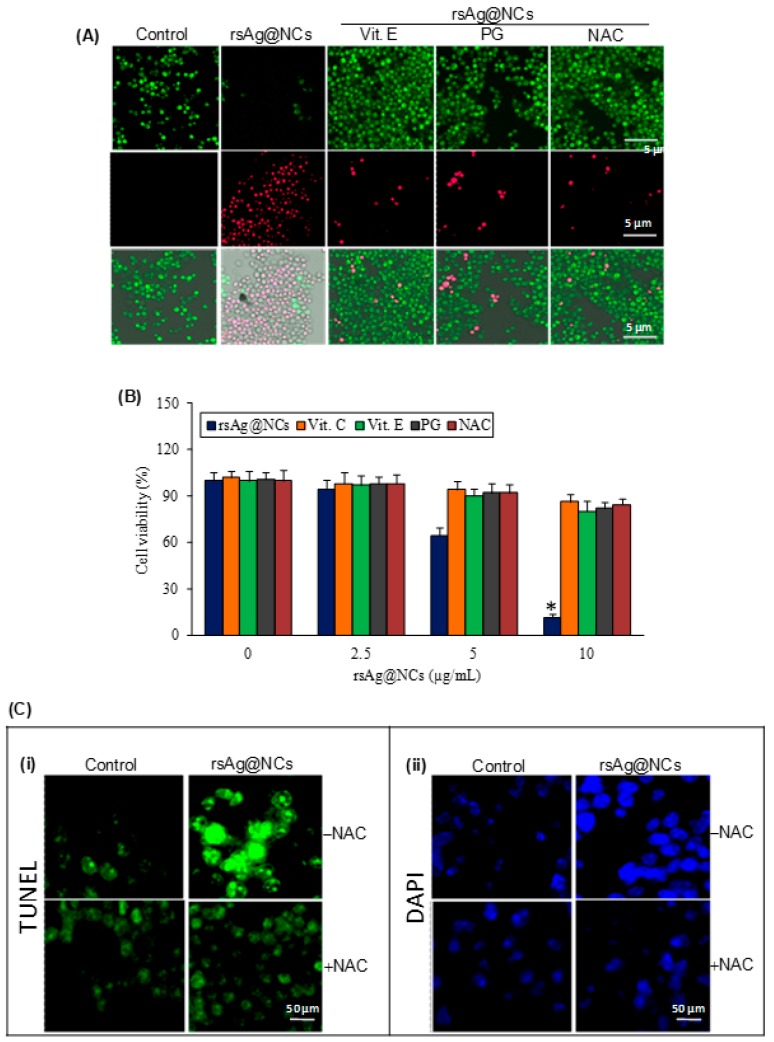
ROS-mediated cell death and apoptosis by rsAg@NCs in FCZ-resistant *C. albicans* NBC099. (**A**) Cells were treated with rsAg@NCs (10 µg/mL) and co-cultured with different free radical scavengers, such as l-ascorbic acid α-tocopherol (Vitamin E; 10 µg/mL), propyl gallate (PG; 10 µg/mL), and *N*-acetyl-l-cysteine (NAC; 10 µg/mL) for 24 h of incubation at 37 °C. Cell viability was determined by using the LIVE/DEAD cell viability kit, following the manufacturer’s protocol (Thermo Fisher). SYTO-9-stained green live cells and PI-stained red cells were analyzed by a Cellinsight CX7 fluorescence microscope. (**B**), Cells were exposed to rsAg@NCs (10 µg/mL) and ROS scavengers: l-ascorbic acid (Vit C; 10 µg/mL), α-tocopherol (Vitamin E; 10 µg/mL), propyl gallate (PG; 10 µg/mL), and *N*-acetyl-l-cysteine (NAC; 10 µg/mL). After 24 h of incubation at 37 °C, cell viability was examined by the 3-(4,5-dimethylthiazol-2-yl)-2,5-diphenyltetrazolium bromide) tetrazolium (MTT) assay. Each reported value represents the mean ± SE from three independent experiments (* *p* < 0.001, compared with the untreated control). (**C**) Cells were treated with rsAg@NCs (10 µg/mL) and co-cultured with a ROS scavenger NAC (10 µg/mL) for 24 h at 37 °C. DNA ends of cells were labelled using (i) the APO BrdU TUNEL Assay Kit, cells were loaded with (ii) DAPI (1 µg/mL), and eventually, cells were analyzed by fluorescence microscopy.

Furthermore, the oxidative stress-protecting effects of l-ascorbic acid (Vitamin C; 5 µg/mL), propyl gallate (PG; 10 µg/mL), *N*-acetyl-l-cysteine (NAC; 10 µg/mL), and α-tocopherol (Vitamin E; 10 µg/mL) were also ascertained. The candidal cells were pretreated with these ROS scavengers followed by rsAg@NCs exposure, and the cell viability was measured. These results revealed that the ROS scavengers protected cells from death from the 10 µg/mL rsAg@NCs, as compared to the cells treated with rsAg@NCs alone (Figure 4A,B). Apoptosis in the presence of NAC was also assessed, and a significant reduction was observed in the apoptosis-inducing potential of the rsAg@NCs (Figure 4C(i,ii)). Overall, the data confirm that the apoptosis and cell death in FCZ-resistant *C. albicans* is induced as a result of ROS generation by rsAg@NCs.

### 3.6. rsAg@NCs Modulate the Expression of Metacaspases in DNA Damage

Metacaspases are distantly related caspase-family cysteine peptidases that are involved in apoptosis in lower eukaryotes. In order to confirm metacaspase activation, untreated and rsAg@NCs-treated cells were incubated with the CaspACE^TM^ FITC–VAD–FMK (in situ marker) and analyzed by a fluorescence microscope. Treated cells showed significant green fluorescence (Figure S18A–C), which was consistent with the positive control treated with H_2_O_2_. In addition, the number of unstained cells appeared in both the untreated control and the NAC treatment (Figure S18A,D). These results suggest that ROS-mediated activation of the metacaspases induces apoptosis in *C. albicans* NBC099.

### 3.7. rsAg@NCs Alter Protein Expression in *C. albicans*

A total of 374 protein spots were detected in both the rsAg@NCs-treated and untreated control samples (Figure S19A,B), of which 252 spots were matched. A total of 103 proteins were differentially expressed, of which 43 proteins were identified by MALDI/TOF-TOF (ABSciex 4800). Among 43 identified proteins, 22 were found to be upregulated and 21 downregulated (Table S1; Figure S20). An analysis of the upregulated proteins found that the highest expression was observed in the Pil1 eisosome (spot id-164), followed by hexokinase (spot id-39), cell division control protein 48 (spot id-13), potential nascent polypeptide associated complex protein (spot id-116), YST protein (spot id-128), GAPDH (spot id-131, 138), and HSP 70 and HSP 104 (spot ids-233, 258). Among the downregulated proteins, the major category was associated with carbon metabolism; more specifically, glycolysis-related proteins like TPI (spot no-86, 87), ENO (spot no-185, 19), PGK (spot no-179), FBA (spot no-143), and TCA and pentose phosphate pathway-related proteins (NADP-GDH (spot no-190), PDC (spot No-17), PGDH (spot no-188), Aconitate hydratase (spot no-253)). Another significant class of down-regulated proteins were related to antiapoptotic activity (Rab GDP dissociation inhibitor; spot id-195) and antioxidative metabolism (thioredoxin peroxidase; spot id-75: Asr2; spot id-105: Formylglutathione hydrolase; spot id-134), virulence (WD repeat protein, spot id-113; potential glycosyl hydrolase, spot id-126, and phosphomannose isomerize, spot id-192). In order to methodically analyze the list of differentially expressed proteins, we scanned for significantly enriched gene ontology (GO) categories. The analysis data showed that differentially expressed proteins were associated with pathways related to glycolysis, energy metabolism, defense response, oxidative stress, mitochondrial function, plasma membrane function, cell death, and apoptosis were significantly altered upon exposure to rsAg@NCs.

### 3.8. rsAg@NCs Regulate the Ras Signaling Pathway

**Figure 5:**
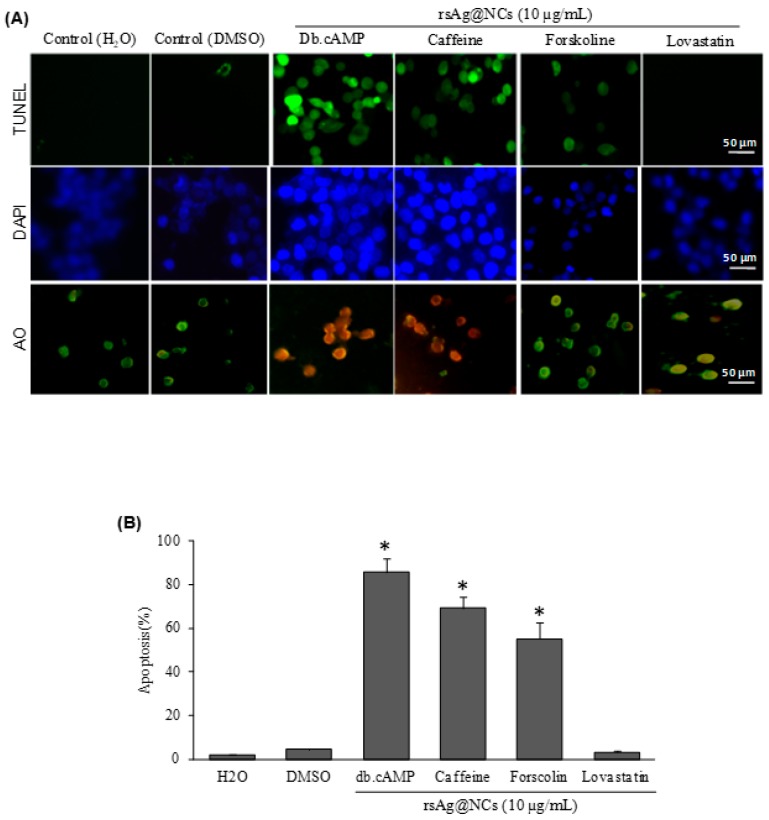
Pharmacological manipulation of the Ras signaling pathway of FCZ-resistant *C. albicans* NBC099. (**A**) NBC099 cells grown on glass cover slides and treated with H_2_O of 0.2% DMSO (as the vehicle control), dibutyryl cAMP (db.cAMP; in water), caffeine (in water), forskolin (in DMSO), or lovastatin (in DMSO). After incubation for 30 min at 37 °C, cells were further treated with 10 µg/mL of rsAg@NCs for 24 h. Immunofluorescence detection of apoptosis was observed by using TUNEL, AO, and DAPI staining assays. For the TUNEL assay, cells were washed and permeabilized. DNA ends of cells were labelled by using the APO BrdU TUNEL Assay Kit, and cells were observed under a fluorescence microscope. Cells were stained with 5 µg/mL and 1 µg/mL of AO and DAPI, respectively. Subsequently, cells were analyzed by fluorescence microscopy. (**B**) To quantify apoptotic cell death, treated or untreated cells were also analyzed by flow cytometry, using the TUNEL assay kit. Water or DMSO (final concentration of <0.2%) was used as a vehicle control for drug treatments. Each reported value represents the mean ± SE from three independent experiments (* *p* < 0.001, compared with the untreated control).

Recently, Phillips et al. reported that the activation of the Ras signaling pathway triggers apoptosis in FCZ-resistant *C. albicans* NBC099 by regulating the expression of anti-apoptotic and pro-apoptotic proteins [45]. To examine whether pharmacological manipulation of the Ras-cAMP pathway could regulate apoptotic cell death, the cells were grown and treated with rsAg@NCs in the presence or absence of dideoxyforskolin and dibutyryl cAMP (db.cAMP). No significant differences could be detected between cells treated with rsAg@NCs in the presence of vehicles: water alone, DMSO or 7 µM dideoxyforskolin. The cells treated with db.cAMP and caffeine were more sensitive to rsAg@NCs, with only 14% and 31% of the cells being viable after incubation for 24 h, compared with 85% and 68% viability in the absence of db.cAMP and caffeine, respectively (Figure 5A,B). The results indicate activation of the Ras-cAMP pathway by rsAg@NCs for accelerating the entry of cells into apoptosis. Two other cAMP-stimulatory agents (5 mM caffeine targeting phosphodiesterase and 7 µM forskolin activating adenylate cyclase) also promoted cell death in response to rsAg@NCs. Co-treatment of rsAg@NCs and 25 mM lovastatin (which blocks Ras farnesylation and localization in the membrane), increased the amount of cell survival, indicating that Ras pathway activation was sufficient to bring about apoptosis at 10 µg/mL of rsAg@NCs.

Lastly, we tested the biocompatibility of rsAg@NCs against mouse embryonic fibroblast 3T3 cells in vitro, using the Alamar blue assay. The obtained data revealed no significance differences as compared to the control, but the potential toxicity of the Cs-AgNPs was observed (Figure S21). A slight decrease in cell viability was noticed when the cells were exposed to a higher concentration of 100 µg/mL, suggesting the non-toxic nature of the rsAg@NCs.

## 4. Discussion

AgNPs are promising and popular materials; their potent antimicrobial properties, good chemical stability, and biocompatibility have already stimulated scientists to develop advanced forms of AgNPs for more efficient biomedical applications. In the present study, we have developed non-toxic silver bionanoclusters, which provide an insight into ROS-mediated apoptosis inducing the activity of rsAg@NCs via the regulation of the Ras-cAMP signaling pathway in FCZ-resistant *C. albicans* NBC099.

Upon interaction with rsAg@NCs, *C. albicans* NBC099 cells underwent a series of proteomic changes that are related to plasma membrane function, glycolysis in the cytoplasm, and the TCA cycle in mitochondria. Two proteins such as Pil1p and Lsp1p form punctuate clusters (eisosomes) on the cytoplasmic surface of the plasma membrane, and they associate with the plasma membrane (PM) via their Bin/amphiphysin/Rvs (BAR) domain proteins. BAR family proteins contribute to a range of cellular functions that are recognized by the membrane and cytoskeletal remodeling, such as membrane curvature and the recruitment of effector proteins [46]. The rsAg@NCs-treated cells exhibited a 9-fold increase in the expression of the Pil1 eisosome component protein (spot id-164), resulting in *C. albicans* plasma membrane remodeling for recruiting other effector protein molecules, probably for initiating apoptosis events. Changes in PM composition further leads to the accumulation of ROS inside the cell. These ROS act as signal molecules for fungi to initiate apoptotic cell death [47]. To continue this process, the fungal apoptosis machinery recruits various metacaspases (Mca1) [48]. Seven substrates of metacaspases (Mca1), including Cdc48, have been identified by Cabezon et al. [49] while studying the apoptosis of *C. albicans* through interactions with murine macrophages. We have found a more than a 4-fold increase in the Cdc48 level (spot id-13) in the rsAg@NCs-exposed cell lysate, which correlates will with the findings of Leger and colleagues [50] in farnesol-induced apoptosis. Cdc48 protein has various roles, including the development of proteolytic pathways involving the proteasome, autophagocytosis-mediated lysosomal degradation, and ribophagy [49]. Thus, Cdc48 may act as either a pro- or an anti-apoptotic signal. A decreased expression of the Rab-GDP dissociation inhibitor protein (RabGDI) was also observed, resulting from exposure to rsAg@NCs, which increased the resistance of cancer cells to the induction of apoptosis by chemotherapeutic agents [51].

Mca1p uses various substrates, which belong to either the family of heat-shock proteins (Hsp70p, Hsp90p, and Hsp104), the translation machinery, or to mitochondria and carbon metabolisms [50]. An increased expression of Hsp70 and Hsp104 in *C. albicans* treated with rsAg@NCs is in good correlation with the findings of Leger et al. [50], supporting the possibility of Mca1 interaction with these chaperon proteins to initiate apoptosis. Studies have shown that survival and cell death are linked to glucose metabolism. Thus, apoptosis is partly dependent on energy status [52]. During the process of apoptosis, glycolytic enzymes have been found to decrease, a clear indication of reduced metabolism and energy demand [49]. We had also found that several enzymes of glycolysis decreased when cells were exposed to rsAg@NCs. Interestingly, some glycolytic enzymes were found to be at elevated levels (e.g., HXK, GAPDH), which suggests that they might be playing a role in inducing apoptotic pathways, as has been demonstrated in studies by Tajimi et al. and Shashidharan et al., [53,54]. The nitrous oxide-targeted GAPDH has been implicated both in H_2_O_2_-induced yeast apoptosis [55] and in the regulation of apoptosis in human cells [56], and we have found increased expression of this enzyme in our study.

Although the protein synthesis machinery was found to be decreased in several studies [49], we have observed the increased abundance of the ribosomal biogenesis protein, along with different elongation factors in the rsAg@NCs-treated cells. A reason for this increase may be an active synthesis of signaling molecule for initiating the cascade of apoptosis. It has been shown that eEF1A is implicated in ROS-induced apoptosis [57]. The process of apoptotic cell death also involves the loss of virulence in *C. albicans*, which is clear from the down-regulated protein expression of the WD-domain repeat-containing protein and glycosyl hydrolase. The WD-repeat protein is required for hyphal development and virulence in *C. albicans* [58]. Glycosyl hydrolase is a potential cell-surface protein in *C. albicans*, which is responsible for adhesion and virulence [59].

A potential antioxidant system determines the fate of the cell. We have measured the reduced levels of thioredoxin peroxidase (Trp), formyl glutathione hydrolase (FGH), and methionine synthase in rsAg@NCs-treated *C. albicans*. These proteins help to maintain cell homeostasis by scavenging ROS, and thereby maintaining reduced glutathione levels inside the cell [60]. Since apoptosis is reported to impair mitochondrial functions, it is likely that these enzymes were reduced during the process of cell death. In the present study, *C. albicans* treated with rsAg@NCs showed more than a two-fold increase in the NAC α subunit. NAC has been reported to regulate protein translocation to the endoplasmic reticulum [61] and mitochondria [62]. Therefore, NAC could play a role, sending a signal to mitochondria for cell death initiation. Cell wall integrity is also an indicator of proper cell functioning. Phosphomannose isomerase (PMI) is an enzyme that catalyzes the interconversion of mannose 6-phosphate (Man-6-P) and fructose 6-phosphate (Fru-6-P) [63]. Fang et al. have shown that PMI activity is essential for viability, and it plays a key regulatory role in both cell wall synthesis and energy production in *Aspergillus fumigates* [63]. In the present study, rsAg@NCs-treated cells also exhibited a reduced level of PMI protein, suggesting a reduction in cell wall integrity and energy production, due to impaired structural integrity. The proteomic data indicate the potential of the rsAg@NCs to cause cellular changes in the metabolic pathways of *C. albicans* which triggers ROS-mediated apoptotic cell death.

The ROS-producing capabilities of metal-based nanomaterials are well known for their implications in cytotoxic responses in various cell types [64]. However, their molecular mechanism for antimycotic activity against FCZ-resistant *C. albicans* NBC099 has not been fully established. A study on *Aspergillus nidulans* indicated that intracellular ROS generation by nanoparticles might be responsible for their antifungal properties, through the formation of apoptosomes [47]. In addition to proteomic analysis, ROS generation due to rsAg@NCs treatment in *C. albicans* was further confirmed. Moreover, ROS-oxidized proteins, particularly iron–sulfur clusters, generate iron ions that are necessary for the Fenton’s reaction to produce ^•^OH. These events appear to be mediated through the transient depletion of the NAD^+^/NADH ratio. This reaction can occur in cells and is, therefore, a possible source of oxidative stress. As expected, the ^•^OH production in the cells increased substantially upon the addition of rsAg@NCs. The measurement of intracellular ^•^OH accumulation in the rsAg@NCs-treated cells enables us to determine whether it plays either a causal role, or is having a secondary effect on the cellular changes that are associated with apoptotic cell death. ROS production was blocked by well-known antioxidants (such as vitamin C and E, PG and NAC), resulting in a significant level protection of rsAg@NCs-treated NBC099 against cell death. These are potent ROS scavengers that have an established means of mitigating the effects of oxidative damage in both eukaryotes and prokaryotes [29]. The results strongly support that ROS induced by rsAg@NCs are key factors responsible for cell death in FCZ-resistant *C. albicans* NBC099. These toxic consequences might also occur due to mitochondrial dysfunction provoked by rsAg@NCs, and these are supported by the data on significant reductions in MMP, OCR, and ECAR. Our observations and conclusions on cell death due to mitochondrial collapse are strongly supported by several previous studies [15,43].

In yeast, the accumulation of deleterious H_2_O_2_ is converted to harmless products by enzymes as part of aerobic metabolism. The mutation of AhpC in *Saccharomyces cerevisiae* decreases the ability to scavenge low levels of H_2_O_2_, compared with the wild-type. AhpC mutants are susceptive to growth inhibition by organic hydroperoxides [65]. Northern analysis showed the upregulation of AhpC after treatment with synthesized rsAg@NCs, providing clues for H_2_O_2_ accumulation. By determining the amount of H_2_O_2_, the ability of rsAg@NCs to induce the level of H_2_O_2_ can be confirmed as being evident from an increased level of the NAD^+^/NADH ratio. Antimicrobial agents have been observed to induce a transient depletion of NADH, stimulate the Fenton reaction, and produce excess ^•^OH formation to damage cells, which suggests a key mechanism of cell death. Many nanomaterials, including iron oxide nanomaterials, AgNPs, and ZnO, also produce ROS to kill fungal as well as bacterial cells [15,66]. These NPs induce differentially expressed genes that are related to detoxification, oxidative/redox stress, drug resistance/sensitivity, and biomolecules stress. The rsAg@NCs modulate the stress response indicated by decreasing the levels of the antioxidant gene pool (GSH, GSH/GSSH, CAT, and SOD) and increasing the levels of oxidative damage markers (MDA content). The ROS-dependent mechanism of action of rsAg@NCs suggests their high toxicity to FCZ-resistant *C. albicans* NBC099 cells.

Apoptosis is a highly regulated cellular cell suicide process, which is crucial for development and differentiation, resulting in the elimination of mutated, unwanted, damaged, or simply dispensable cells without an inflammatory reaction [67]. Here, it is evident that the rsAg@NCs induced apoptosis in FCZ-resistant *C. albicans* NBC099, which can be further confirmed by DNA and nuclear fragmentation, immunofluorescence, and TUNEL assays. A range of nanomaterials has apoptosis activation potential via oxidative stress signaling [15,29]. To test this hypothesis, cells were treated with rsAg@NCs and the activation of classical metacaspase signaling pathways was monitored. Treated cells exhibited activation of these pathways, again confirming the major involvement of oxidative stress signaling in the induction of apoptotic cell death in NBC099. The observations and conclusions on apoptotic cell death due to mitochondrial dysfunction, oxidative stress, and apoptosis related pathways activation, are well-supported by earlier studies [29,68]. Phillips et al. reported that the Ras-cAMP pathway plays an important role in the apoptosis response of *C*. *albicans* as induced by oxidative stress-inducing agents such as acetic acid and H_2_O_2_ [45]. In the present study, the enhanced activation of the Ras-cAMP pathway in NBC099 may be due to oxidative stress and mitochondrial dysfunction by the treatment of rsAg@NCs. The involvement of the Ras signaling pathway in cell death through pharmacological manipulation is also established. Artificial Ras activation accelerates the rate at which cells become apoptotic. In the present study, it is also confirmed that pharmacological disruption of Ras-cAMP signaling inhibits the rate of entry into apoptosis. The present study is the first of its kind, and provides initial clues that biogenic rsAg@NCs have the potential to induce cell death and oxidative stress, which results in apoptosis in FCZ-resistant *C. albicans* NBC099. The results clearly show that rsAg@NCs activate the Ras-cAMP signaling pathway for triggering apoptosis in *C. albicans*, which was previously not known.

## 5. Conclusions

This work demonstrates that non-toxic rsAg@NCs trigger apoptosis in FCZ-resistant *C. albicans* NBC099 by targeting ROS-dependent Ras signaling. The downstream effectors of the Ras-mediated cell-death response in NBC099 have yet to be identified. Our biogenic rsAg@NCs target fungal Ras-cAMP signaling, and might be used to enhance the efficacy of traditional antifungal therapies by blocking the expression of antiapoptotic stress responses or activating proapoptotic proteins, perhaps turning rsAg@NCs into more attractive antifungal agents. Promoting the onset of fungal apoptosis could improve the outlook for patients with recurrent infections or life-threatening systemic diseases caused by FCZ-resistant *C. albicans*.

## Supplementary Materials

The following are available online at http://www.mdpi.com/2218-273X/9/2/47/s1.
