# Supplementary material for: Non-Toxic and Ultra-Small Biosilver Nanoclusters Trigger Apoptotic Cell Death in Fluconazole-Resistant Candida albicans via Ras Signaling
Source: Biomolecules. 2019 Jan 30;9(2):47. doi: 10.3390/biom9020047 (PMC6406502; doi:10.3390/biom9020047)
Supplement: Supplementary file 1 [file biomolecules-09-00047-s001.pdf]

SUPPLEMENTARY INFORMATION

Non-toxic and ultra-small biosilver nanoclusters trigger apoptotic cell death in fluconazole-resistant *Candida albicans* via Ras signaling

Supplementary Methods

Preparation of plant extract

Usnea longissima was collected from the Govind Wildlife Sanctuary, Uttarkashi district, Uttaranchal, India in May 2013. Shade dried sample was powdered (40-mesh) and stored in polythene bags at 4 ^{0}C. Aqueous extract of *U. longissima* was prepared by boiling powdered sample (100 g) with 1 L distilled water (DW) for 60 min, followed by sonication for 15 min. The supernatant was obtained through centrifugation at 10000 rpm for 8 min and dried in a freeze dryer (Labconco, USA). Extract yield was obtained to be 5.7 g. To extract maximum polyphenolic compounds, 5 g of extract was redissolved in 200 mL of DW, subjected for sonication for 20 min, filtered, and eventually fractionated with 500 mL of ethyl acetate. Finally, ~0.9 g extract was obtained by evaporating solvent under rotary evaporator (BUCHI, USA) at 30 ^{0}C. The resulting dry extract (AEU) was used for the biosynthesis of Ag-NCs.

Estimation of polyphenolic contents

TPC and TFC of AEU were measured as described elsewhere by Singh et al. (*69*)and Singh et al. (*70*), respectively. For quantification of TPC, AEU (1 mg/mL; 1 mL), Folin–Ciocalteau's reagent (1 N; 1.0 mL) and sodium carbonate (20%; 2 mL) were added subsequently. The test mixture was left at room temperature for 15 min and maintained to 25 mL with DW. The absorbance of the test mixture was measured at $A_{725\,nm}$ using UV–Vis spectrophotometer (Thermo Fisher, USA) and results were expressed as GAE/g. For determination of TFC, aluminium chloride (2%; 5 mL) was mixed with WEP solution (1.0 mg/mL; 1 mL). Absorption readings at $A_{415\,nm}$ were taken after 10 min against a blank sample consisting of 5 mL AEU and 5 mL DW without aluminium chloride. TFC was estimated using a standard curve of quercetin (0–100 µg/mL), and results were presented in terms of QE/g.

HPLC analysis

AEU (0.2 g) was acid hydrolysed with 50% ethanol (10 mL) containing 1.2 N HCl by refluxing on a water bath for 2 h and fractionated with ethyl acetate (3 x 50 mL). Ethyl acetate fraction was dried under a vacuum rotary evaporator. The extract was dissolved in HPLC grade methanol (1.0 mg/mL), filtered through sterile 0.22 µM filter membrane (Millipore, India) and applied for qualitative and quantitative analysis using Shimadzu LC-10A (Kyoto, Japan) HPLC instrument. The HPLC equipped with a dual-pump LC-10AT binary system (Shimadzu, Kyoto, Japan), a UV detector SPD-10A (Shimadzu, Kyoto, Japan), and a Phenomenex Luna RP, C_{18} column (4.6 × 250 mm). Separation was achieved with acetonitrile/water containing 1% acetic acid as mobile phase in a linear gradient program, started with 15% acetonitrile, changing to 35% in 15 min and finally to 50% in 40 min. Results were obtained by comparing the peak area of AEU's chemicals and their standards at λ_{max} 254 nm (*71*).

Reducing potential (RP) of AEU

RP was determined using a slightly modified ferric reducing-antioxidant power assay (*70*). Briefly, 1.0 mL of AEU was mixed with 2.5 mL of phosphate buffer (0.15 M, pH 6.5) and 2.5 mL of 1% potassium ferricyanide. The mixture was incubated at 40 ^{0}C for 20 min. The reaction was terminated by adding 2.5 mL of 10% trichloro acetic acid and centrifuged at 7000 rpm for 10 min. The upper layer (2.5 mL) was diluted with an equal volume of DW. Finally, 0.5 mL of 0.1% ferric chloride was added. After 10 min, the absorbance was measured at A_{700} nm.

SEM analysis

Untreated or rsAg@NCs-treated candidal cells were washed with PBS and fixed overnight in 2.5% glutaraldehyde in phosphate magnesium buffer. Cells were postfixed for 2 h in 2% osmium tetroxide for 15 min, washed twice with PBS, and stained with 1% aqueous solution of uranyl acetate for 30 min. After two further washings, cells were dehydrated in 95% and 100% ethanol, subsequently. Hydrated cells were exposed to propylene oxide for 2x10 min. Cells were infiltrated for 1 h in 1:1 propylene epoxy embedding material (Epon) mixture and in fresh Epon for overnight. After polymerization at 65 ^{0}C for 45 h, ultrathin sections were cut and stained with uranyl acetate and lead citrate. Sections were examined under an SEM to detect apoptotic cell death.

Quantification of OCR and ECAR

The mitochondrial OCR and ECAR were also quantified using an XF Assay Kit in the XF Extracellular Flux Analyzer (Seahorse Bioscience). OCR and ECAR are reported in pmoles/min.

References

69. Singh, B.N.; Singh, B.R.; Singh, R.L.; Prakash, D.; Sarma, B.K.; Singh, H.B. Antioxidant and anti-quorum sensing activities of green pod of Acacia nilotica L. *Food Chem. Toxicol.* **2009**, *47*, 778–786, doi.org/10.1016/j.fct.2009.01.009.
70. Singh, H.B.; Singh, B.N.; Singh, S.P.; Nautiyal, C.S. Solid-state cultivation of Trichoderma harzianum NBRI-1055 for modulating natural antioxidants in soybean seed matrix. *Bioresour. Technol.* **2010**, *101*, 6444–6453, doi.org/10.1016/j.biortech.2010.03.057.
71. Singh, B.N.; Singh, B.R.; Singh, R.L.; Prakash, D.; Singh, D.P.; Sarma, B.K.; Upadhyay,G.; Singh, H.B. Polyphenolics from various extracts/fractions of red onion (Allium cepa) peel with potent antioxidant and antimutagenic activities. *Food Chem. Toxicol.* **2009**, *47*, 1161–1167, doi.org/10.1016/j.fct.2009.02.004.

SUPPLEMENTARY FIGURES

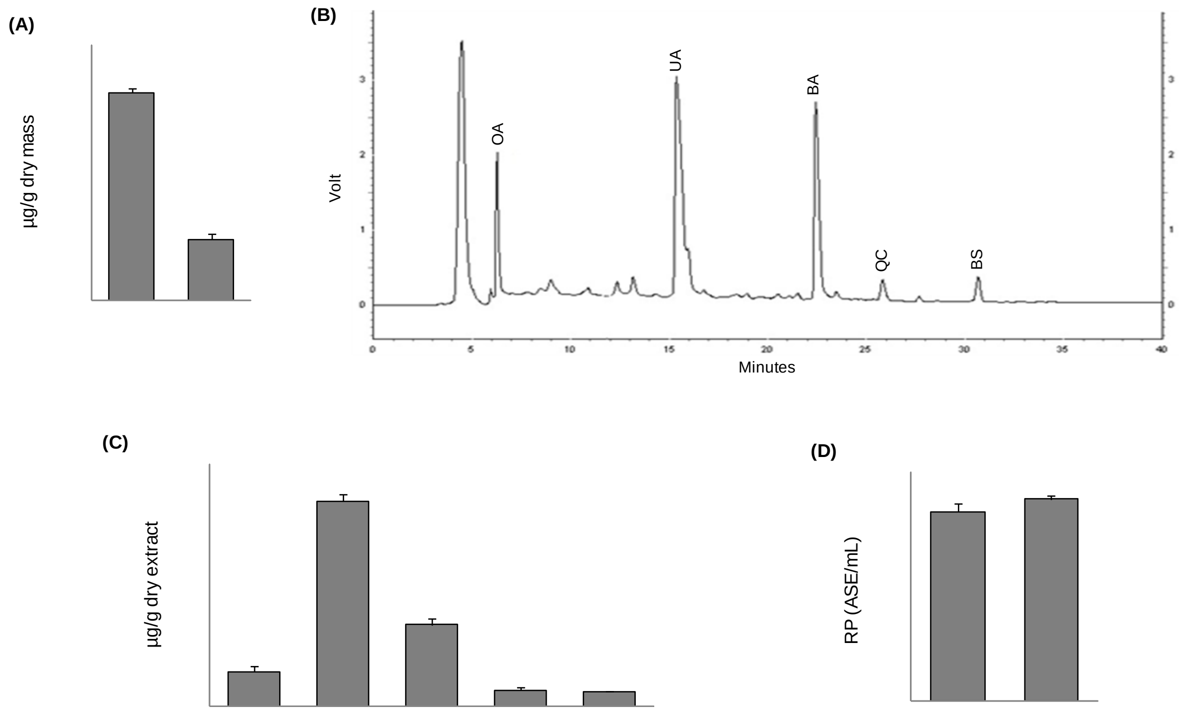

Figure S1. Phytochemicals and reducing potential (RP) of an aquoeus extract of *U. longissima* (AEU). (A) Total phenolic content (TPC) and total flavonoid content (TFC) of AEU expressed as µg gallic acid equivalent (GAE)/g dry extract and µg quercetin equivalent (QE)/g, respectively. (B) HPLC chromatograms of WEP and identification of compounds were obtained by comparison of the peak (λmax 254 nm) of the sample with that of standards. OA, oleanolic acid; UA, usnic acid; BA, barbatinic acid; QC, quercetin; BS, beta-sitosterol. (C) Contents of identified polyphenols of AEU. (D) RP of AEU, expressed as ascorbic acid equivalent (ASE)/mL, 1 mM = 1 ASE) is inversely proportional to RP. BHT; butylated hydroxytoluene

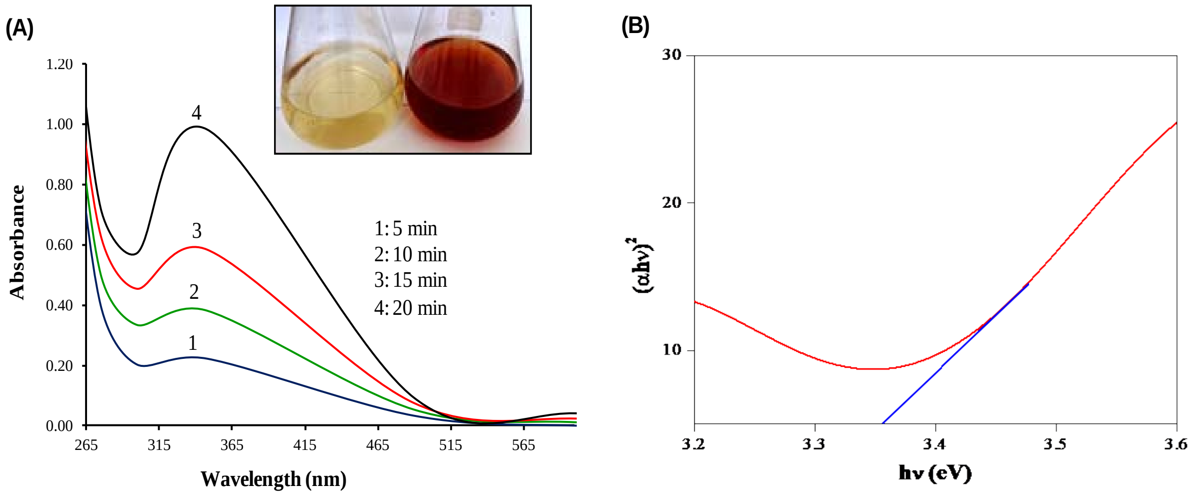

Figure S2 Biosynthesis and characterization of rsAg@NCs. (A) A solution of silver acetate (3 mM) was challenged with AEU (2 mg/mL), followed by adding polypheol oxidase (PPO; 5 units/mL) in a reaction culture flask and recorded UV-vis spectra of the rsAg@NCs at various time intervals at 340 nm. The insert shows a digital image of the color changes during the NCs formation using AEU as a potent reductant. (B) Tauc plot depicts the energy band gap of rsAg@NCs which was found to be ~3.3 eV.

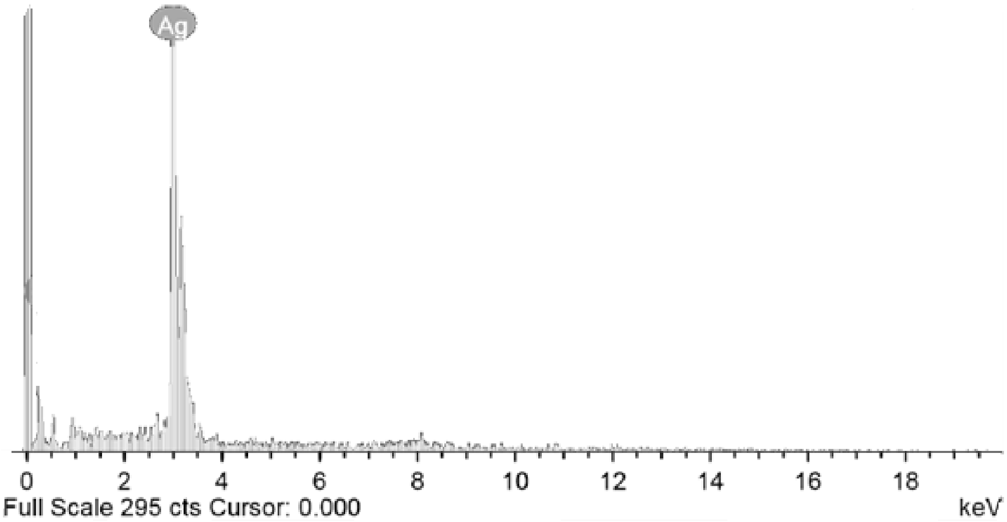

Figure S3. Characterization of rsAg@NCs. EDAX spectrum reveals the elemental composition of synthesized rsAg@NCs.

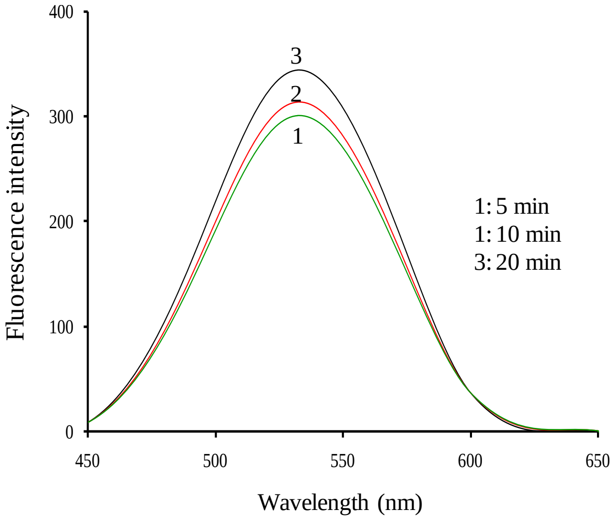

Figure S4. Fluorescence emission spectra of the rsAg@NCs at different time intervals. The photoluminescence behaviour of rsAg@NCs suggests the energies and dynamics of photogenerated charge carriers as well as on the nature of the emitting states. The emission spectra have broadband with a maximum 532 nm when excited at 350 nm.

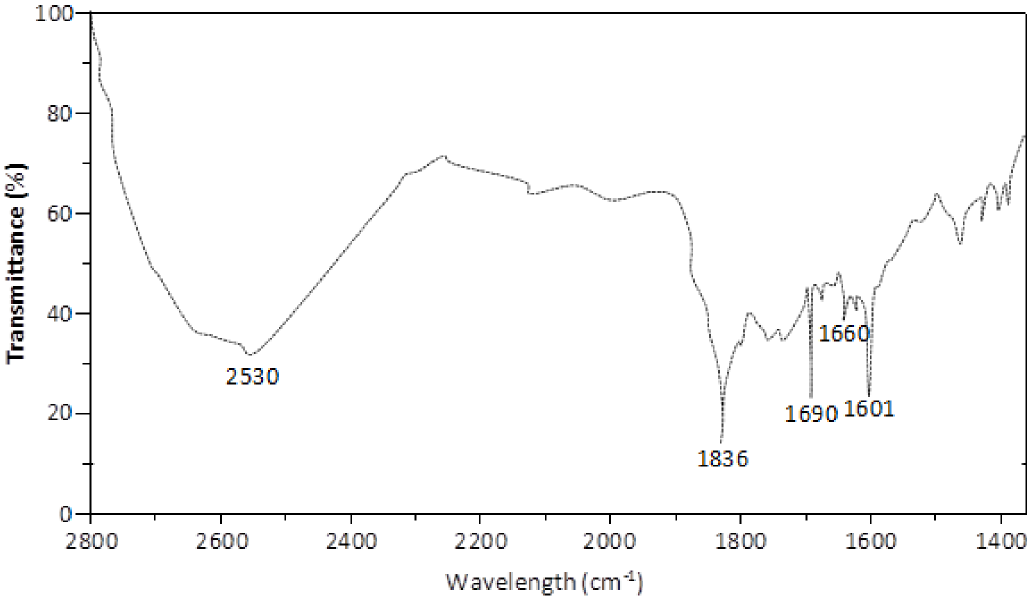

Figure S5. FTIR spectrum of the rsAg@NCs. The obtained spectrum shows different major peak positions at 2503, 1836, 1690, 1660, and 1601 cm^{-1} showing the interaction between NPs and phytochemicals of AEU. The peaks located at 1660 and 1601 cm^{-1} could be assigned to C=O stretching or amide bending, and the broad and intense peak at 2530 cm^{-1} corresponds to OH stretching vibrations of phenol/carboxylic group. Plant quinones contain free oxygen groups with the neutral charge that may increase the formation of NC of AgNPs due to altering the zeta potential of the NPs. Therefore, it could be inferred that the polyphenolics and quinones of *U. longissima* are responsible for synthesizing and stabilizing the rsAg@NCs.

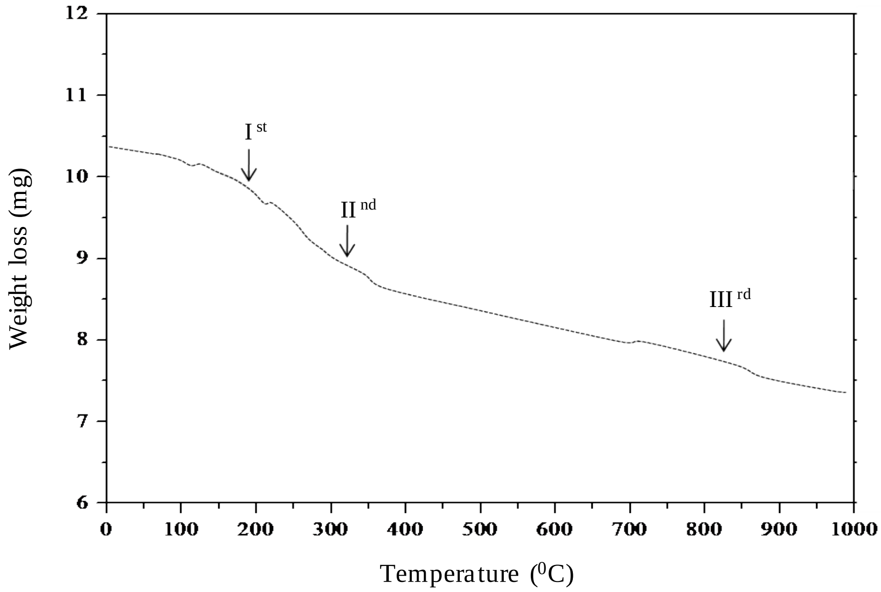

Figure S6. TGA graph illustrating the thermal behaviour of rsAg@NCs.

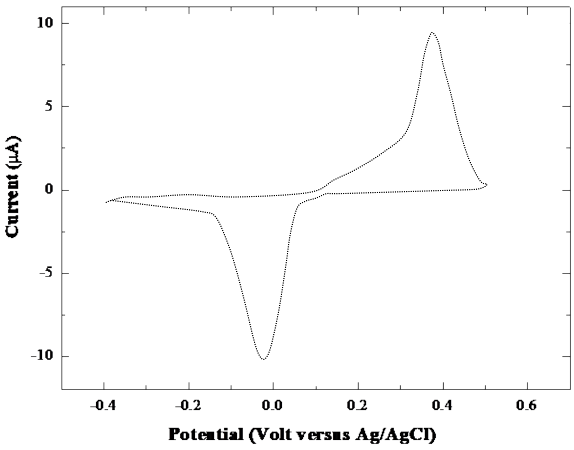

Figure S7. CV analysis revealing the redox potential of rsAg@NCs

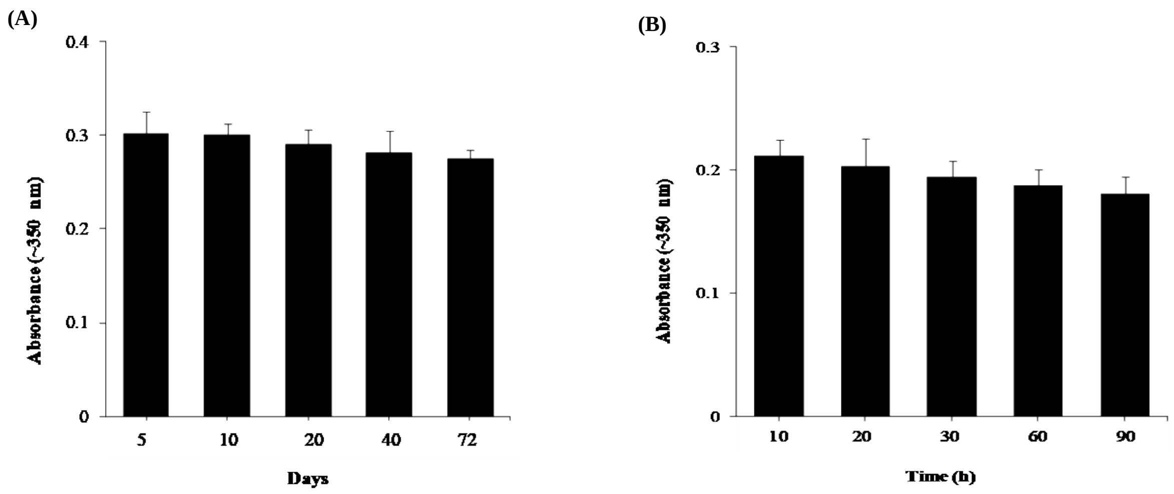

Figure S8. (**A**) Stability of the rsAg@NCs up to 72 days at room temperature and (B) in the culture medium (SG broth) at 37 ^{0}C for 90 h. Bar graphs are showing the time dependent change in the absorbance of rsAg@NCs at 350 nm. The values represent the mean ± SE from three independent experiments.

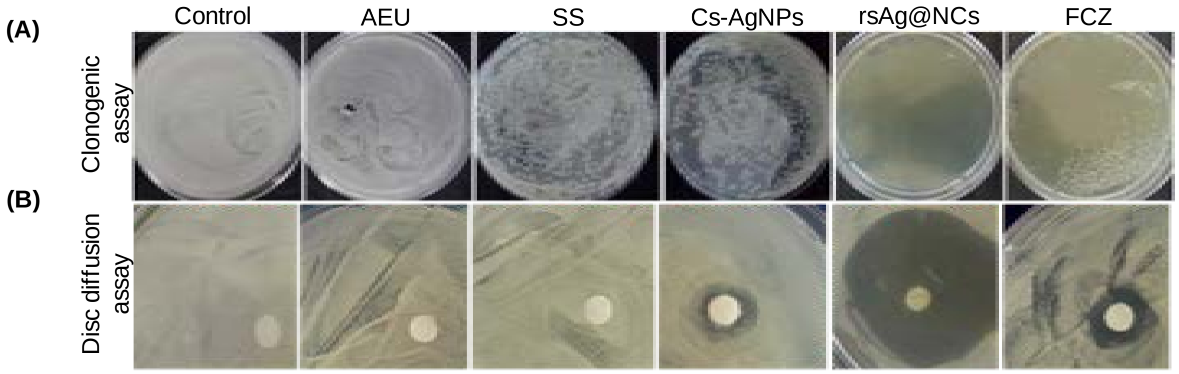

Treatment	Colony forming unit (cfu)/mL **(A)**	Zone of inhibition (ZOI) (mm) **(B)**
Control (DW)	Uncountable	0.0
Aqueous extract of *U. longissima* (AEU; 200 µg/mL)	Uncountable	0.0
Sodium selenite (SS; 10 µg/mL)	$10x10^9$	0.0
Chemically synthesized AgNPs (Cs-AgNPs; 10 µg/mL)	$6x10^7$	4.7 ± 0.2
rsAg@NCs (10 µg/mL)	Nil	19.2 ± 0.5
Fluconazole (FCZ; 60 µg/mL)	$1x10^6$	7.3 ± 0.4

Figure S9. Anticandidal activity of the rsAg@NCs. (A) For clonogenic assay: the active culture of FCZ-resistant *C. albicans* NBC099 ($1x10^{10}$ cells/mL) was centrifuged, washed the pallets and then resuspended in PBS. Aliquots (100 µL) of suspended cells were spread on SG agar plates, containing various treatments: AEU (200 µg/mL), SS (10 µg/mL), Cs-AgNPs (10 µg/mL), FCZ (60 µg/mL), and rsAg@NCs (10 µg/mL). The plates were incubated at 37 ^{0}C for 24 h and examined for the formation of colonies. (B) The cell suspension was also used for agar disc diffusion assay and spread uniformly on SG agar plates. Above treatments were loaded onto the pre-sterilized filter paper discs. After 24 h of incubation at 37 ^{0}C, the zone of inhibition (ZOI) was calculated. The inserted table shows colony forming unit (cfu)/mL and ZOI values of indicated treatments.

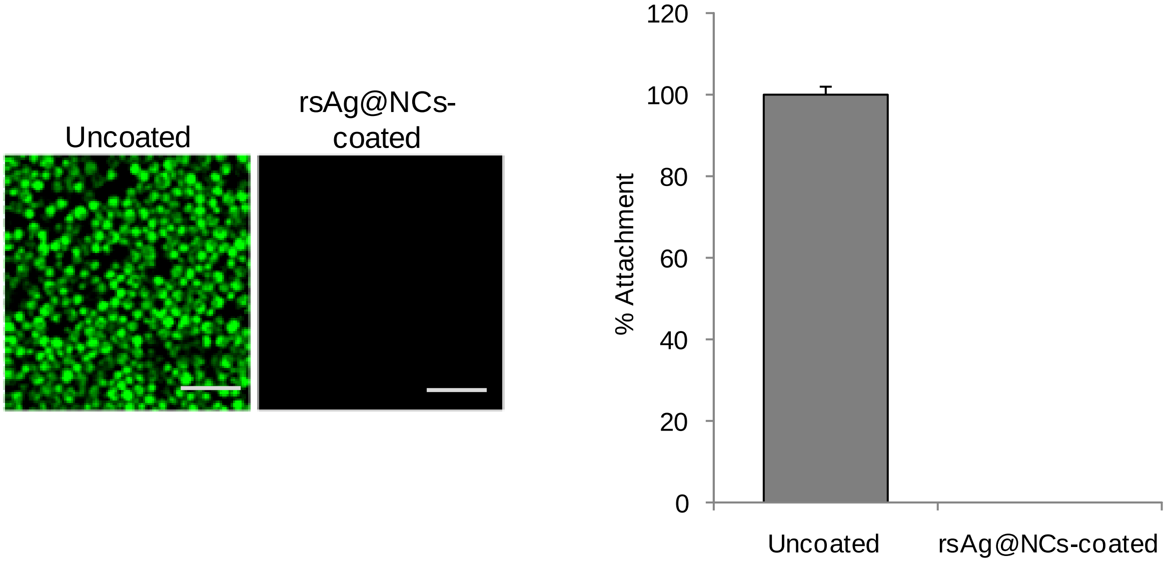

Figure S10. Hydrogel coatings by spin-coating with rsAg@NCs onto glass surfaces and examined capacity to resist *C. albicans* cells attachment on these surfaces using a fluorescence SYTO-9 green stain viability assay. The values represent the mean ± SE from three independent experiments. Scale bars, 50 μm

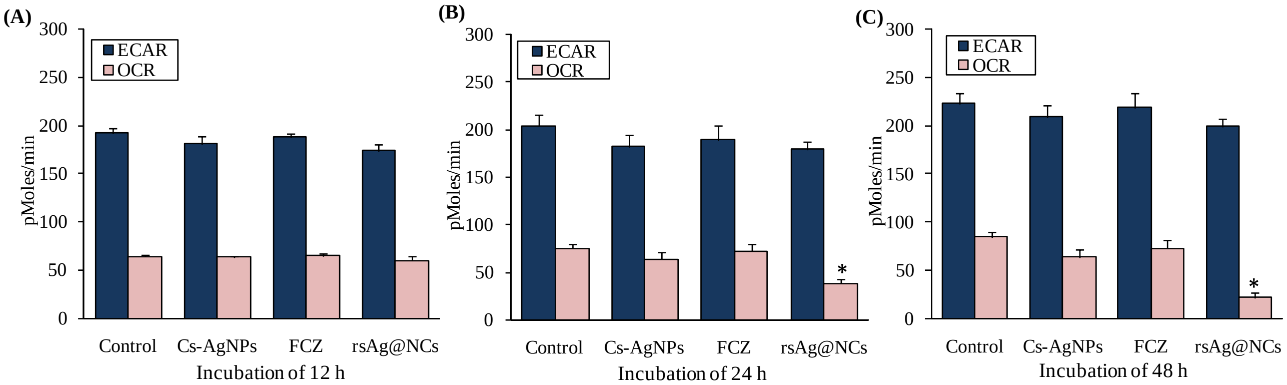

Figure S11. Effect of rsAg@NCs on mitochondrial function of *C. albicans*. The mitochondrial oxygen consumption rate (OCR) and extracellular acidification rate (ECAR) of treated *C. albicans* cells with Cs-AgNPs (10 µg/mL), FCZ (60 µg/mL), and rsAg@NCs (10 µg/mL) for different time intervals such as (A) 12 h, (B) 24 h and (C) 48 h of incubation. The rsAg@NCs treatment induced a burst of mitochondrial OCR, which was followed by a drastic drop after 24 h. Representative bar plots from three independent experiments were shown, and numerical values represent means ± SE (*P < 0.001, compared with untreated control).

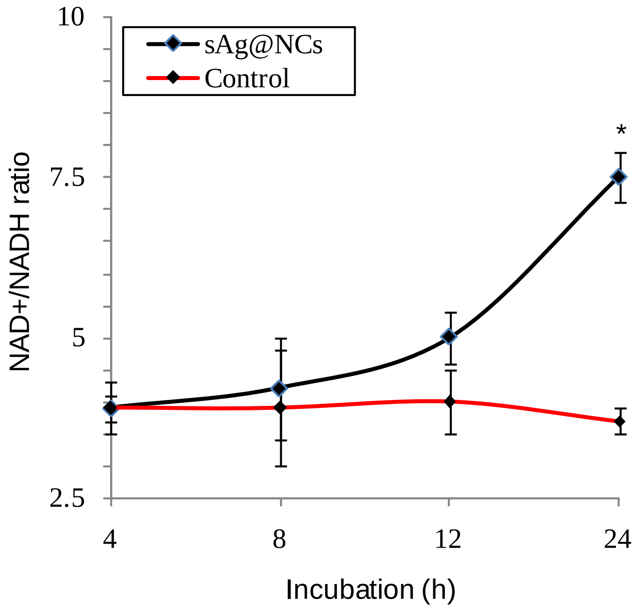

Figure S12. The ratio of NAD^+ to NADH (NAD^+/NADH) for 10 μg/mL rsAg@NCs-treated and untreated *C. albicans* at different time intervals. The values represent the mean ± SE from three independent experiments (**P* < 0.001, compared with untreated control).

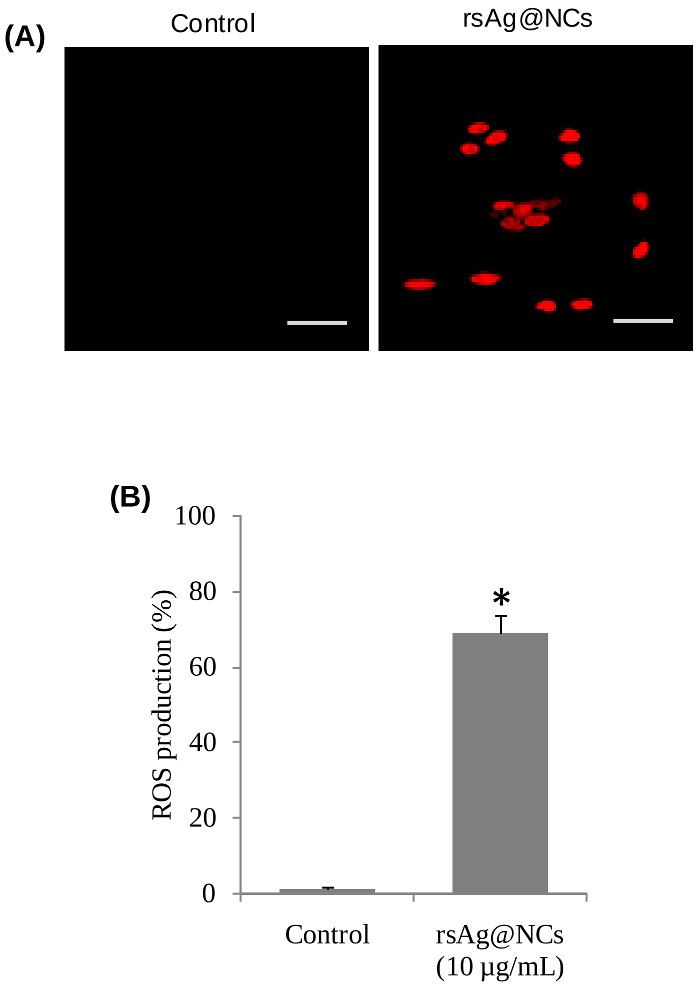

Figure S13. Intracellular ROS accumulation by rsAg@NCs. (A) FCZ-resistant *C. albicans* NBC099 cells were seeded on glass cover slides and exposed to rsAg@NCs (10 µg/mL) for 24 h at 37 ^{0}C. After washing twice with PBS, cells were incubated with 5 mM H_2DCFDA and examined using a fluorescence microscope (Scale bar: 5 µm). (B) Cells were also grown into 96-well microtiter plates with the above treatments and then cells were loaded with H_2DCFDA. ROS accumulation was subsequently analysed by measuring fluorescence intensity at 540 nm using a microplate reader. Each reported value represents the mean ± SE from three independent experiments (*$P < 0.001$, compared with untreated control).

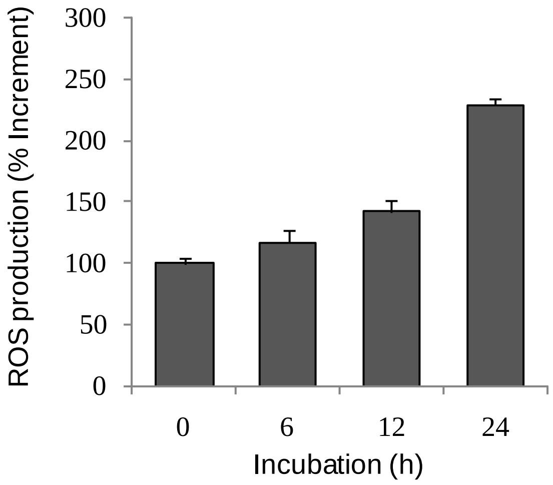

Figure S14. Time-dependent accumulation of intracellular ROS was assessed by spiking rsAg@NCs (10 μg/mL)-treated cells with dihydrorhodamine (DHR) 123 (5 μg/mL) for 30 min before the end of each experiment. Cells were then harvested, washed twice with ice-cold PBS and examined by flow cytometry. The values represent the mean ± SE from three independent experiments.

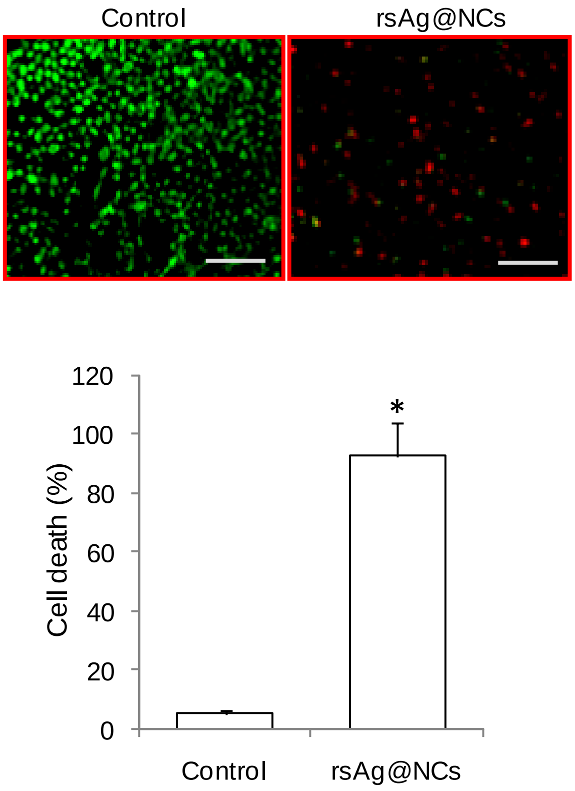

Figure S15. Inhibition of cell viability by rsAg@NCs. Cells were seeded on glass cover slip for 12 h in the absence or presence of rsAg@NCs (10 µg/mL) in SG broth medium at 37 ^{0}C. After 24 h of incubation, cells viability was assessed using LIVE/DEAD cell viability staining kit. Quantitative cell viability data obtained from image analysis. The values represent the mean ± SE from three independent experiments (*P < 0.001, compared with untreated control). Scale bars, 50 µm.

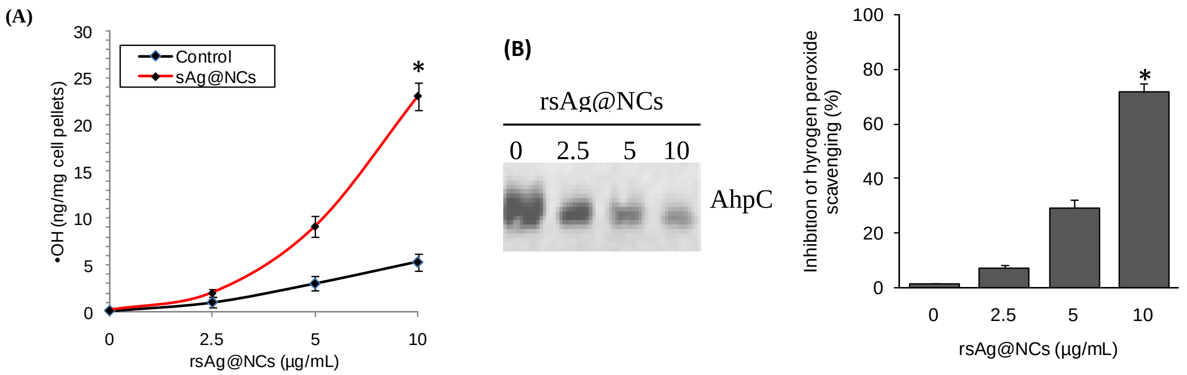

Figure S16. Impact of rsAg@NCs on ROS-related biological processes in *C. albicans*. (A) Generation of hydroxyl free radical (•OH) accumulation, when cells treated with various concentrations of rsAg@NCs (0, 2.5, 5, and 10 µg/mL) for 24 h at 37 ^{0}C. (B) Detection of AhpC level using the Northern blotting technique in untreated or treated cells for 24 h with indicated concentrations of rsAg@NCs. (C) Hydrogen peroxide (H_2O_2)-scavenging ability of untreated or treated cells with different concentrations of rsAg@NCs. We added H_2O_2 (a final concentration of 1.5 mM) to equal yeast concentrations of rsAg@NCs-treated or untreated cells, incubated them for 30 min, and determined the H_2O_2 concentration. The scavenging ability is indicated by the percent reduction of the H_2O_2. Representative bar plots from three independent experiments were shown, and numerical values represent means ± SE (*P < 0.001, compared with untreated control).

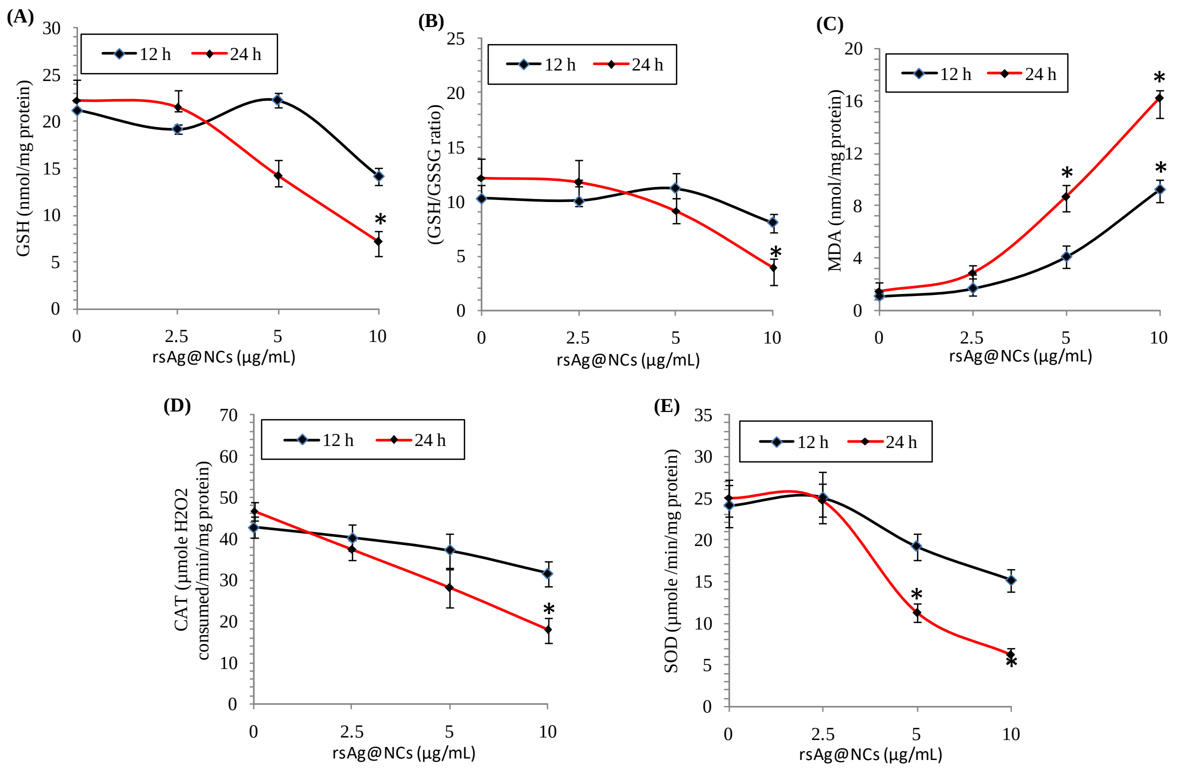

Figure S17. Effect of rsAg@NCs on an endogenous antioxidant system of *C. albicans*. Cells were exposed to various concentrations of rsAg@NCs for 12 h and 24 h. Then, cells were harvested and lysed in cell lysis buffer [20 mMTris-HCl (pH 7.5), 150 mM NaCl, 1 mM Na_2EDTA, 1% Triton and 2.5 mM sodium pyrophosphate]. Collected supernatant was subjected for examination of oxidative-stress biomarkers such as (A) total glutathione (GSH) content, (B) GSH/GSSH ratio, (C) formation of malondialdehyde (MDA; a product of LPO), (D) catalase (CAT) activity, and (E) superoxide dismutase (SOD) activity. Each reported value represents the mean ± SE from three independent experiments. (*P < 0.001, compared with untreated control).

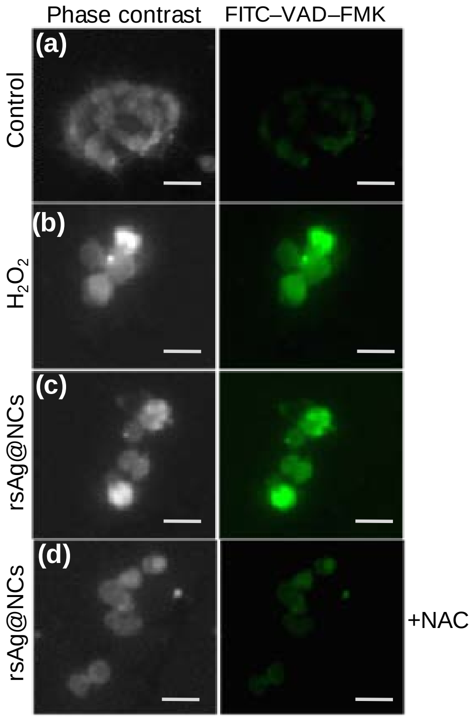

Figure 18. Effect of rsAg@NCs on the activity of metacaspase in FCZ-resistant *C. albicans* NBC099. (A) The rsAg@NCs-treated cells for 24 h were washed, resuspended in 200 μL staining solution containing 10 μM of CaspACE FITC–VAD–FMK and incubated for 30 min at room temperature in the dark. Prepared cells were analysed with a fluorescence microscope. (a) control; (b) 3 mM H_2O_2 (oxidative stress)-treated cells; (c) rsAg@NCs (10 μg/mL)-treated cells; (d) NAC (10 μg/mL)-treated cells. Scale bars, 50 μm.

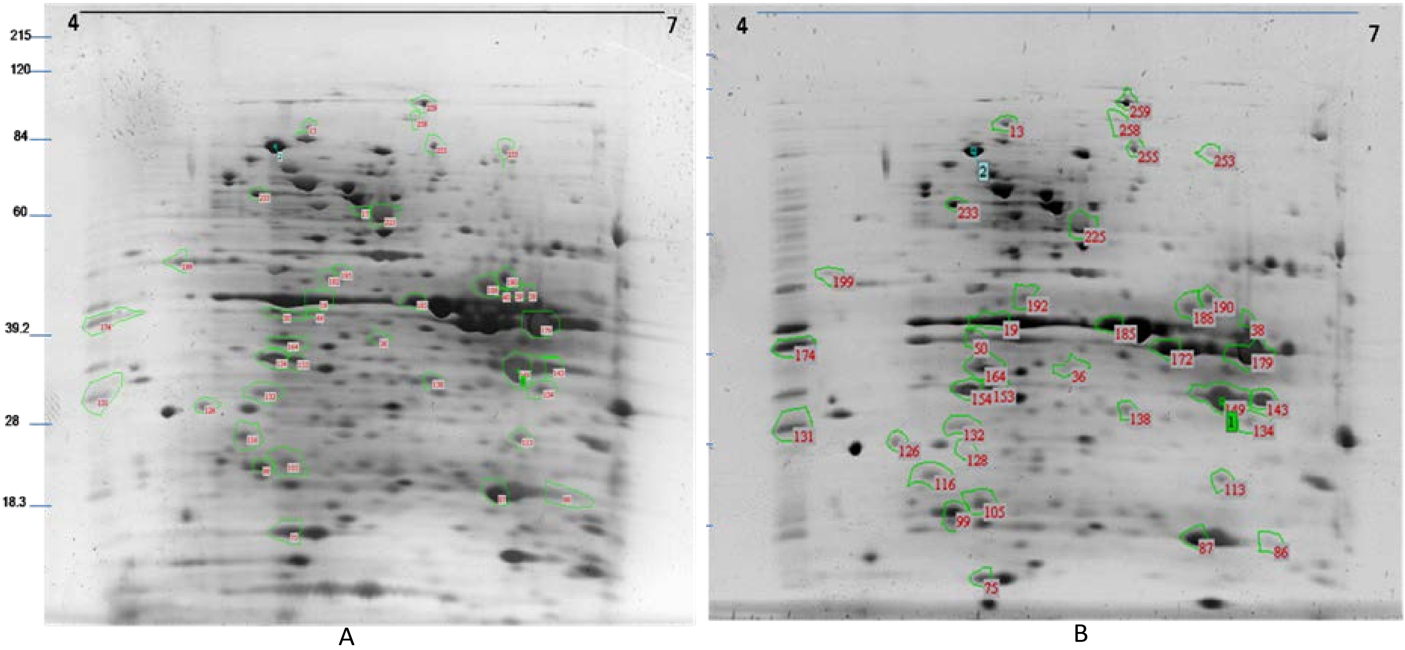

A B

Figure S19. Two-dimensional (2D) gel electrophoresis. Silver stained representative gels of *C. albicans* proteins of (a) Control and (b) after interaction with 10 µg/mL of rsAg@NCs. The gels reveal the differentially expressed proteins of *C. albicans* NBC099.

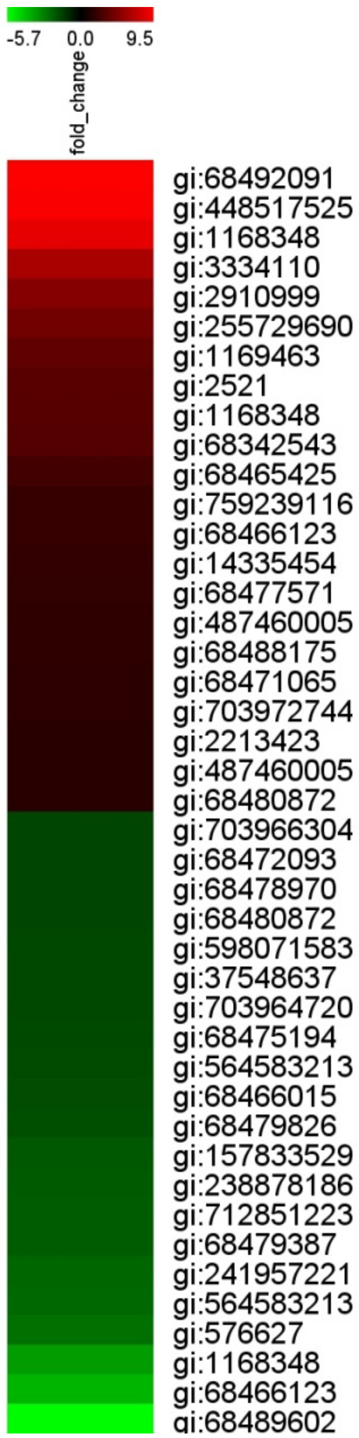

Figure S20. Heatmap analysis of the rs-Ag@NCs-treated proteins of *C. albicans* NBC099. Heatmap showing differentially expressed proteins of *C. albicans* treated with 10 μg/mL of rsAg@NCs. The downregulated expression is indicated by green shading, upregulated expression by red shading.

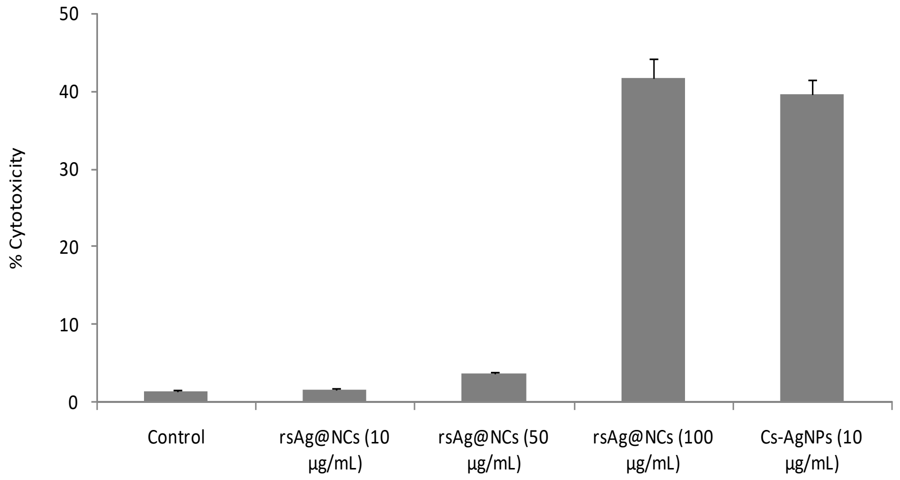

Figure S21. Impact of the rsAg@NCs on cell viability of 3T3 cells. The cells were cultured in 96-well flatbottom microplate with the indicated concentrations of rsAg@NCs and Cs-AgNPS, for 48 h. The cellular viability was assessed using an Alamar blue (AB) assay. The pipette out 0.4 mL of AB dye (Thermo Fisher, USA) was diluted with 10 mL of PBS. Hundreds of microlitters dye solution was added into each well and incubated for 4 h at 37 ^{0}C. The viability of cells was measured at 570 nm. Cell viability data indicate that the rsAg@NCs is highly non-toxic and biocompatible upto 50 µg/mL concentration, while Cs-AgNPs show potential toxicity. DW was used as a control. There were three technical replicates, and the cell viability was given as a mean value. Error bars indicate the S.E.

Table S1: List of differentially expressed proteins of *C. albicans* treated with rsAg@NCs

Match Id	Fold change	Protein description	Accession number	Mascot score	Matched peptides	Sequence coverage	MW/PI	Function
13	+4.32	Cell division control protein [*Candida tropicalis* MYA-3404] *CDC-48*	gi|255729690	412	10(2)	16%	91.2/4.9	Regulated for cell division
36	+1.79	Potential ubiquinol cyt-c reductase core protein 2 [*Candida albicans* SC5314] *UQCRC2*	gi|68488175	93	1(1)	3%	39.5/5.45	Involved in oxidative phosphorylation
38	+3.63	Probable NADPH dehydrogenase hypothetical *EBP1*	gi|1169463	399	8(4)	28%	46.1/5.98	Oxidative stress
39	+9.49	Hexokinase-like protein [*Candida albicans* SC5314] *GLK1*	gi|68492091	143	2(1)	7%	52.4/ 5.86	Plays role in apoptosis
40	+6.36	6-Phosphogluconate dehydrogenase, decarboxylating hypothetical *6PGD*	gi|3334110	166	3(2)	7%	57.1/6.14	Involved in pentose phosphate pathway
49	+8.63	Alcohol dehydrogenase 1 hypothetical *ADH1*	gi|1168348	411	8(4)	29%	37.2/6.02	Converted glucose to ethanol and CO_2
50	+3.28	Alcohol dehydrogenase 1 hypothetical *ADH1*	gi|1168348	311	7(1)	23%	37.2/6.02	Alcohol dehydrogenase (NAD) activity
116	+2.51	Potential nascent polypeptide-associated complex alpha subunit [*Candida albicans* SC5314] *EGD2*	gi|68465425	321	6(3)	43%	19.4/4.7	Involved in transcription regulation
128	+5.07	YST protein [*Candida albicans*] hypothetical *ystA*	gi|2910999	361	5(3)	24%	28.0/5.12	Ribosome-associated protein: controlled biofilm formation
131	+1.82	Type I glyceraldehyde-3-phosphate dehydrogenase [*Escherichia coli*] *GAPDH1*	gi|487460005	564	9(5)	29%	35.9/6.61	Involved in oxidation-reduction process

132	+1.75	Likely cytosolic ribosomal acidic protein P0 [*Candida albicans* SC5314] *RPP0*	gi|68471065	152	3(1)	13%	33.2/4.74	Damage response
136	+2.11	Ribosome biogenesis protein [*Phialophora attae*] *BMS1*	KPI43237.1	55	1(1)	1%	13.3/5.99	Act as a molecular switch during maturation of the 40S ribosomal subunit in the nucleolus
138	+1.59	Type I glyceraldehyde-3-phosphate dehydrogenase [*Escherichia coli*] *GAPDH1*	gi|487460005	314	6(3)	24%	35.9/6.61	Involved in oxidative phosphorylation
149	+2.07	Fructose-bisphosphate aldolase [*Candida tropicalis*] *CTRG_00211*	gi|68466123 ACF33499.1	394	5(3)	18%	39.3/5.69	Involved in gluconeogenesis
153	+1.87	E1 beta subunit of the pyruvate dehydrogenase (PDH) complex, putative [*Candida dubliniensis* CD36]	XP_002420029.1	410	9(2)	26%	41.5/5.37	Involved in glycolysis
154	+1.74	Transaldolase [*Candida albicans* P57072] *MBM_09374*	gi|703972744	571	11(5)	38%	35.8/4.96	Required for metabolism
164	+9.38	Pil1 eisosome component [*Candida orthopsilosis* Co 90-125]	gi|448517525	276	7(1)	25%	34.2/4.93	Involved in plasma membrane remodelling
199	+3.19	Probable translation elongation factor EF-1 alpha [*Candida albicans* SC5314] *EF-1 alpha*	gi|68342543	206	5(2)	10%	50.4/9.11	Implicated in oxidative stress-induced apoptosis
225	+1.54	Pyruvate decarboxylase [*Candida albicans* P75010] *PDC11*	gi|68480872	801	13(4)	33%	62.7/5.39	Participated in glycolysis
233	+1.59	Heat shock protein 70 [*Candida albicans*] *HSP70*	gi|2213423	360	5(4)	13%	66.5/5.16	Biomarker of oxidative damage
258	+1.94	Heat shock protein 104 [*Candida albicans*]	gi|14335454	242	7(1)	10%	100.1/5.42	Chaperon proteins to

		HSP104						initiate apoptosis
259	+3.32	Translation elongation factor 3 [*Candida albicans*] *TEF-3*	gi|2521	53	2(0)	1%	117.5/5.52	Involved in protein biosynthesis
17	-1.57	Pyruvate decarboxylase [*Candida albicans* P75010] *PDC11*	gi|68480872	750	11(6)	30%	62.7/5.39	Participated in glycolysis
19	-2.33	Enolase, partial [*Candida albicans*] *ENO1*	gi|564583213	794	12(7)	38%	47.0/5.54	Controlled the yeast-to hypha conversion by regulating glycosis and gluconeogenesis
75	-1.75	Likely thioredoxin peroxidase [*Candida albicans* SC5314] *TPX*	gi|68479826	394	7(4)	35%	21.9/4.98	Involved to scavenge ROS and apoptosis inhibition
86	-1.55	Triosephosphate isomerase [*Candida albicans*] *TPI1*	CAB77631.1	54	2(0)	8%	26.8/5.74	Required for carbohydrate biosynthesis
87	-1.60	Triosephosphate isomerase [*Spathaspora passalidarum* NRRL Y-27907] *TPI1*	gi|598071583	423	7(4)	14%	26.7/ 5.35	Glycolysis
99	-1.60	29 kDa IgE-binging protein [*Candida albicans*]	gi|37548637	144	3(2)	19%	25.9/ 4.79	Allergic marker
105	-1.57	Asr2 protein [*Candida orthopsilosis* Co 90-125] *ASR2*	XP_003869127.1	93	2(1)	9%	27.7/ 5.43	Involved in stress response
113	-2.32	WD repeat protein (G-beta like protein) :orthologue of mammalian RACK1, putative; [*Candida dubliniensis* CD36]	gi|241957221	141	3(1)	16%	34.7/ 6.07	Required for hyphal development and virulence

126	-1.72	Potential glycosyl hydrolase [*Candida albicans* SC5314] *GH*	gi∣68466015	541	8(4)	25%	33.8/ 4.56	Involved in carbohydrate metabolic process
134	-2.06	S-formylglutathione hydrolase [*Candida albicans* L26] *SFGH*	gi∣712851223	229	4(2)	13%	34.1/ 6.05	Required for quenching ROS
143	-4.06	Fructose-1,6-bisphosphate aldolase, putative [*Candida dubliniensis* CD36] *FBA1*	gi∣68466123 XP_002419830.1	231	5(1)	18%	39.3/ 5.69	Controlled growth and viability
172	-3.50	Alcohol dehydrogenase 1 [*Candida albicans*] *ADH1*	gi∣1168348	320	7(2)	23%	37.2/ 6.02	Required for cell growth
174	-2.52	Alcohol dehydrogenase [*Candida albicans*] *ADH1*	gi∣576627	379	6(3)	23%	37.2/ 5.76	Involved in biofilm formation
179	-5.65	Phosphoglycerate kinase [*Candida albicans* P76067] *PGK*	KHC32675.1	739	16(6)	41%	45.2/ 6.07	Involved in glycolysis
185	-1.68	Enolase, partial [*Candida albicans*] *ENO1*	gi∣564583213	762	13(6)	34%	47.2/ 5.54	Controlled the yeast-to hypha conversion by regulating glycosis and gluconeogenesis
188	-1.60	6-Phosphogluconate dehydrogenase, decarboxylating [*Candida albicans* P94015] hypothetical *6PGD*	gi∣703964720	477	9(4)	21%	54.6/ 5.75	Involved in pentose phosphate pathway
190	-1.54	NADP-specific glutamate dehydrogenase [*Candida albicans* GC75] *GDH-NADP*	gi∣703966304	966	16(8)	41%	49.9/ 5.73	Required for biosynthesis of glutamate
192	-1.97	Chain A, Phosphomannose Isomerase [*Candida albicans*] *1PMI*	gi∣157833529	102	4(0)	9%	48.9/ 5.16	Involved in cell survival

195	-2.02	rab GDP-Dissociation inhibitor [*Candida albicans* WO-1]	gi|238878186	68	3(0)	10%	50.4/ 5.09	Required for resistance to apoptosis
253	-2.08	Likely mitochondrial aconitate hydratase [*Candida albicans* SC5314] *ACO1*	gi|68479387	532	12(4)	19%	84.6/ 5.96	Tricarboxylic acid cycle
255	-1.65	Likely cobalamin-independent methionine synthase [*Candida albicans* SC5314]	gi|68475194	230	6(2)	8%	85.7/ 5.44	Required for growth

"**+**" and "**-**" signs show the up and down-regulation respectively as compared to their respective untreated controls
